# Melanin-like nanoparticles: advances in surface modification and tumour photothermal therapy

**DOI:** 10.1186/s12951-022-01698-x

**Published:** 2022-11-19

**Authors:** Luyao Tian, Xia Li, Haixia Ji, Qing Yu, Mingjuan Yang, Lanping Guo, Luqi Huang, Wenyuan Gao

**Affiliations:** 1 Tianjin Key Laboratory for Modern Drug Delivery & High-Efficiency, School of Pharmaceutical Science and Technology, Tianjin University, Tianjin, 300193 China; 2 National Resource Center for Chinese Materia Medica, Academy of Chinese Medical Sciences, Beijing, 100700 China

**Keywords:** Melanin, Nanoparticle, Cancer, Surface modification, Photothermal therapy

## Abstract

Currently, tumor treatments are characterized by intelligence, diversity and personalization, but the therapeutic reagents used are often limited in clinical efficacy due to problems with water solubility, targeting, stability and multidrug resistance. To remedy these shortcomings, the application of multifunctional nanotechnology in the biomedical field has been widely studied. Synthetic melanin nanoparticles (MNPs) surfaces which contain highly reactive chemical groups such as carboxyl, hydroxyl and amine groups, can be used as a reaction platform on which to graft different functional components. In addition, MNPs easily adhere to substrate surface, and serve as a secondary reaction platform to modify it. The multifunctionality and intrinsic biocompatibility make melanin-like nanoparticles promising as a multifunctional and powerful nanoplatform for oncological applications. This paper first reviews the preparation methods, polymerization mechanisms and physicochemical properties of melanin including natural melanin and chemically synthesized melanin to guide scholars in MNP-based design. Then, recent advances in MNPs especially synthetic polydopamine (PDA) melanin for various medical oncological applications are systematically and thoroughly described, mainly focusing on bioimaging, photothermal therapy (PTT), and drug delivery for tumor therapy. Finally, based on the investigated literature, the current challenges and future directions for clinical translation are reasonably discussed, focusing on the innovative design of MNPs and further elucidation of pharmacokinetics. This paper is a timely and comprehensive and detailed study of the progress of MNPs in tumor therapy, especially PTT, and provides ideas for the design of personalized and customizable oncology nanomedicines to address the heterogeneity of the tumor microenvironment.

**Graphical Abstract:**

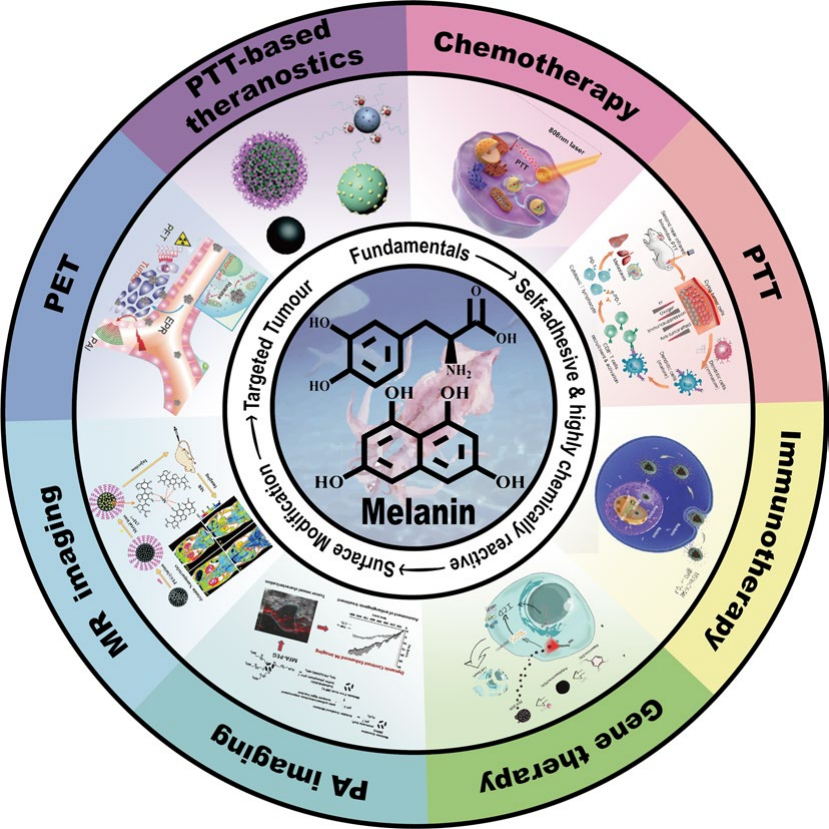

## Introduction

Due to high rates of morbidity and mortality, cancer is a major public health concern. There are expected to be approximately 1,918,030 new cancer cases and 609,360 cancer deaths in the United States by 2022 [1]. Recently, there have been significant advancements in cancer research and treatment. Traditional cancer treatments include chemotherapy, surgery, radiotherapy, and proton therapy [2, 3], with chemotherapy remaining the most prevalent method due to its high efficacy in treating both primary and metastatic tumors. However, high doses and repeated dosing are required to effectively deliver the drugs to the tumor due to the lack of targeting. Consequently, cytotoxic chemotherapeutic drugs inevitably cause tissue damage while killing cancer cells [4, 5]. Therefore, how to precisely deliver drugs to tumor tissues for targeted therapy is an issue that requires immediate scientific attention. In recent years, antitumor nanomedicines have demonstrated promising application and development prospects for enhancing the efficacy of cancer treatment, particularly in the diagnosis and treatment of tumors based on nanoparticles (NPs) [6]. Blood vessels in tumor regions have irregular shapes, curvature, and holes compared to those in normal tissues as a result of the abnormal proliferation of tumor tissues and other factors. Due to their enhanced permeability and long retention effect (EPR effect), nanomedicines with a size of 20–200 nm can effectively enter and accumulate in the tumor area by penetrating aberrant blood vessels within the tumor and accumulating there [7–9]. Nanomedicines can protect the loading components, improve drug accumulation and intratumoral penetration in tumor tissues, and reduce adverse effects on normal tissues, which indicates great application and development potential in cancer therapy [10, 11]. In addition to the monofunctional nanoparticles prepared by conventional methods, multifunctional NPs have been designed that show excellent results in anticancer therapy [12–14]. Multifunctional nanoparticles (MFNPs) can be surface-modified to provide targeted delivery or even co-delivery of contrast agents and multiple therapeutic agents (e.g., genetic material and chemotherapeutic agents) [15, 16]. MFNPs with a variety of functionalities have been fabricated on the basis of various materials, including polymeric nanoparticles, mesoporous nanoparticles, magnetic nanoparticles, and gold nanoparticles [17].

Surface modification of materials has been one of the research topics of greatest interest in the field of materials science, as surface coatings can protect internal materials from a harsh external environment. In addition, surface coatings can serve as secondary reaction platforms to control surface properties or even impart new functions to materials [18]. Existing established methods for surface modification, such as layer-by-layer assembly, hydrolysis, chemical conjugation, and plasma treatment, are frequently complicated and time-consuming processes that are not always applicable to all surfaces. Therefore, the challenge of discovering an effective and simple coating method for any surface remains unresolved [19], although it is at the centre of efforts in this field. Among the many available materials, melanin, a biomolecule from living organisms, is receiving increasing attention due to its natural biocompatibility, biodegradability and multifunctional physiological role in the organism [20].Melanins are pigments with different structures and origins produced through the oxidation and polymerization of tyrosine in animals or phenolic compounds in lower organisms [21], including (i) melanins of narrow natural origin, such as *eumelanin* and *pheomelanin*; ii) *Pyomelanins*, produced by microorganisms; and (iii) melanin synthesized from natural precursors [22]. Melanin including natural and synthetic melanin-like nanoparticle surface which contains highly reactive chemical groups such as carboxyl, hydroxyl and amine groups, can be used as a reaction platform for grafting different functional components [23]. Melanin is typically present in the mucous membranes, eyes, hair, skin, and brain medulla of living organisms and has a variety of biological functions, such as UV resistance, photosensitization, metal ion chelation, intrinsic photoacoustic properties, and ultrafast, thermal relaxation [12–18]. In addition, melanins contain an abundance of catechol structures, allowing them to react with molecules containing amino and sulfhydryl termini via Schiff base and Michael addition reactions, respectively. Therefore, they can facilitate the surface functionalization of nanomaterials with other biomolecules for specific biological applications and act as drug delivery systems for imaging-guided tumor therapy by piggybacking on tumor therapeutics via- bond interactions [24–26]. The properties of melanin have been the subject of extensive research, and nanomaterials derived from melanin, such as polydopamine (PDA), have been studied for numerous applications, including material coatings, wastewater purification, energy storage and conversion, and biomedical applications [27–29]. Several outstanding reviews of melanin-like substances have also been published. Wang et al*.* (2018), for instance, provide an overview of the various applications of PDA in tumor-targeted drug delivery systems and a comprehensive account of drug release behavior in drug-loaded PDA nanoparticles [30]. Cheng et al*.* review the accomplishments of PDA platform-based materials in advanced nanomedicine and surface modification from 2016 to 2019 [31]. Liu et al*.* present a methodical, exhaustive, and well-developed review of melanin nanoparticles (MNPs) in biomedicine [32]. In 2020, Hong et al*.* briefly summarized the use of melanin-based nanomaterials in cancer treatment [33]. Since melanin pigments are natural polyphenolic pigments, Shaimaa et al*.* summarized the platform of MNPs as a platform for heavy metal removal agents, peroxynitrite scavengers and PTT anticancer agents based on their antioxidant properties [34]. Mariana et al*.* focused on the biomedical applications of MNPs, with a review of their applications in photovoltaics, photoconductivity and photoacoustics (PA), including implications and theranostics [35]. In 2022, Lasmina et al*.* briefly summarized the applications of functionalized pigment-like nanoparticles in medical oncology, but their list of applications is not exhaustive and comprehensive [36]. Nonetheless, there are a significant number of literature reviews on surface modification based on PDA that are manifestly not representative of melanin. In addition, they do not provide an exhaustive and well-established review of surface modification-based MNPs in oncological nano-delivery systems. A comprehensive review of MNPs in the context of oncology therapy that can guide the clinical translation of melanin-like nanoparticles based on cancer therapy is urgently needed.

This review will discuss recent advancements in MNP research, including MNPs of natural origin, namely, *eumelanins* and *Pheomelanins*, and synthetic melanin-like nanoparticles represented by polydopamine (PDA). regarding surface modification and oncology therapy especially photothermal therapy (PTT) based on the most recent melanin-like research findings. A brief description of the structure and polymerization mechanisms of melanin is followed by a summary of MNP surface modification strategies. We conclude with a comprehensive summary of emerging nanoparticles for cancer therapy especially PTT based on melanin-like surface modifications (Table 1). We hope that this review will facilitate the application of melanin-like surface modifications in oncology nanodelivery systems, provide researchers with access to effective antitumor approaches through a variety of melanin-like based design platforms, and facilitate the clinical translation of these approaches.

## Preparation and polymerization mechanism of MNPs

**Fig. 1:**
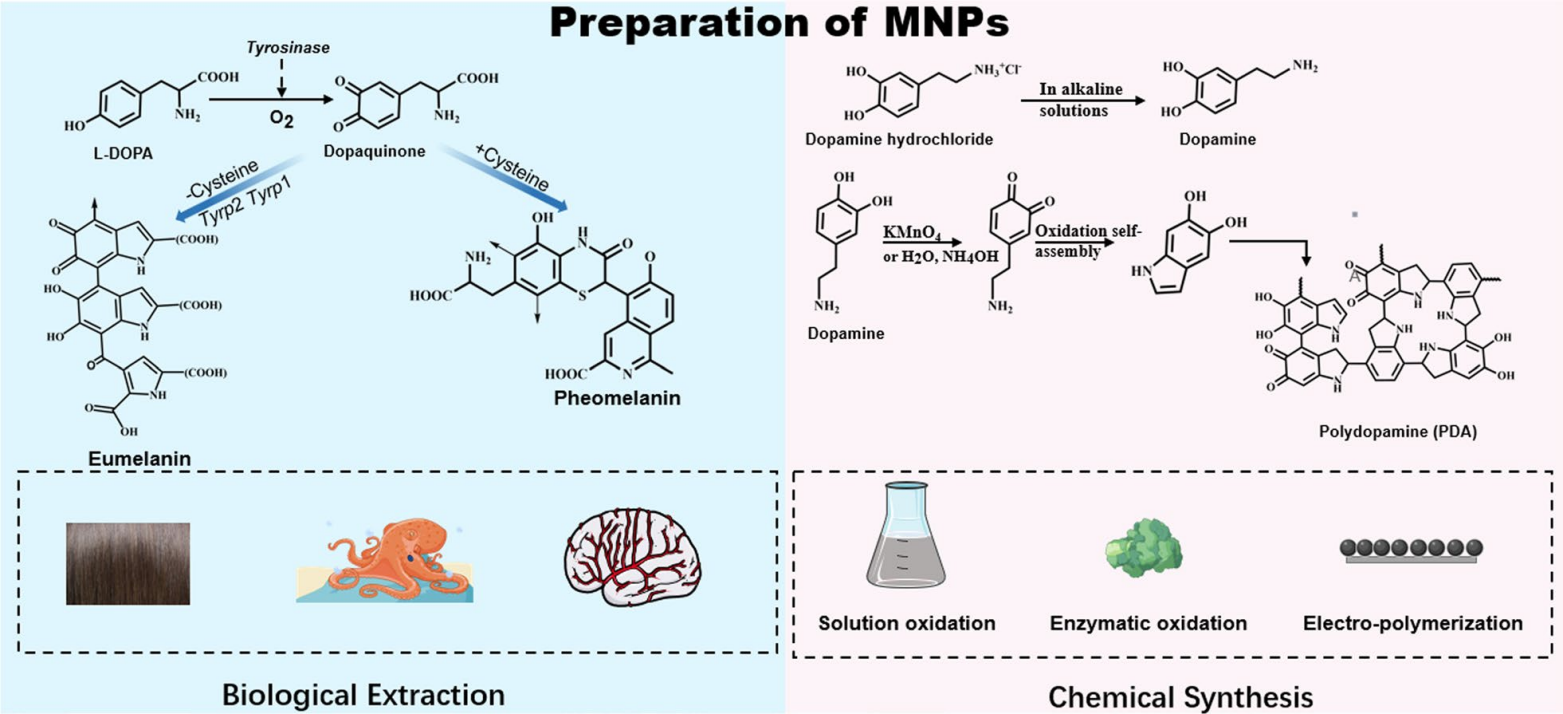
Preparation and synthesis pathways of MNPs. Left: purification natural melanin from natural sources and then employing existing nanotechnology to prepare natural MNPs. The diagram also shows the biosynthesis pathways of *eumelanin* and *pheomelanin*. Right: chemical oxidation dopamine

Melanin can be divided into melanin of natural origin in a narrow sense, melanin produced by microorganisms and melanin synthesized from natural precursors [21, 22, 37]. Therefore, MNPs are typically prepared in one of two ways: extracting and purifying natural melanin from natural sources, or employing existing nanotechnology to prepare natural MNPs or chemically oxidizing dopamine hydrochloride (or sometimes L-DOPA) to synthesize PDA (or poly(L-DOPA)) NPs (Fig. 1)

Due to the ability to control their size, their uniform particle size distribution, and their good dispersion in water and biological media, chemically synthesized MNPs are more commonly used. There are three main methods to prepare synthetic melanin, including solution oxidation, enzymatic oxidation and electropolymerization. Solution oxidation is the most commonly used method and involves adding an oxidant at an alkaline pH. In brief, L-DOPA or dopamine hydrochloride is added through pH-induced oxidative polymerization in alkaline solutions (pH > 7.5) under mild and basic conditions to form MNPs. Few scholars have studied (poly(L-DOPA)) NPs due to the lack of satisfactory and facile synthetic strategies for the structural and functional control of MNPs. Therefore, the melanin-like NPs referred to this paper, also called synthetic MNPs are PDA NPs. Enzymatic oxidation and electropolymerization methods are not often used due to the complex steps required to purify functional enzymes and the fact that they are only deposited on the surface of materials.

Despite the widespread use of PDA in materials and biomedical applications, few authors have studied the molecular mechanism and precise chemical structure of its polymerization [28, 38–42]. Lee et al*.* first proposed PDA as a homopolymer constructed from indole (1, 2) or from different indole fractions in various oxidation states [4]. In 2010, Yu et al*.* calculated the structure of polydopamine as a diindole structure by simulating infrared spectroscopy, LUMO energy, etc. [39]. In 2012, Hong and Daniel et al*.* demonstrated that the formation of PDA is not covalently linked as previously proposed by researchers but based on the self-assembly of oligomeric oxidation products of 5,6-dihydroxyindole (DHI) [28, 40]. In 2013, Jürgen et al*.* developed a structural model of PDA based on results derived from several analytical methods, including ^13^C CPPI MAS NMR spectroscopy, ^1^H MAS NMR spectroscopy, and high-resolution ES( +)-MS, which consisted of a mixture of different oligomers with varying degrees of (un)saturation of indole units and open-chain dopamine units [41]. In 2018 Hong et al*.* demonstrated that cationic π-stacking plays an important role in the formation of PDA [42]. According to previous scholarly studies, two PDA polymerization mechanisms are now generally accepted: (1) The oxidation-polymerization mechanism: the catechol of DOPA is oxidized to dopamine quinone (DAQ) by an oxygen-driven collodion structure. The amine group is deprotonated and then cyclized intramolecularly via a 1,4-Michael addition reaction to the non-adhesive DHI, which can self-polymerize into (dopamine)_2_/DHI dimers or other oligomers by forming its own covalent bonds, followed by the disproportionation of catechol with phthalic acid to form semiquinone radicals that cross. This polymerization mechanism is similar to the biosynthetic pathway for melanin [30]. (2) Covalent and non-covalent polymerization-based binding mechanisms. Hong et al*.* observed using high-performance liquid chromatography-mass spectrometry (HPLC–MS) that a large amount of unpolymerized dopamine was stably encapsulated, and only a small amount was released into the matrix [42]. This demonstrates that the dopamine polymerization process, in addition to covalent self-polymerization, can form the (dopamine)2/ DHI trimer, consisting of two dopamine molecules tightly linked to one DHI molecule via T-type interactions.

Synthetic MNPs are simple to prepare but still lack a large amount of clear evidence of their broad biocompatibility for biomedical applications. Alessandro et al*.* investigated the biocompatibility of synthetic DHI melanin and found that synthetic eumelanin supports normal murine embryonic stem cell (ESC) growth without impairing proliferation and survival [43]. Christopher et al*.* explored synthetic melanin biotoxicity in vitro and in vivo and found that PC12 cells cultured on melanin thin films grew faster and that melanin implants were almost completely degraded in vivo [44]. However, in the study by Marina et al*.* verified that high concentrations of melanin killed fibroblasts (NIH3T3); however, after a 48-h incubation period, low concentrations of melanin promoted cell proliferation. They also found synthesized in dimethylsulfoxide (D-Mel) promoted cell proliferation better than melanin synthesized in water (W-Mel) in vitro [45]. Natural melanin is produced by biochemical reactions in vivo. Tyrosine is first hydroxylated in mammals by tyrosine hydroxylase to obtain L-DOPA, which can then be decarboxylated to form dopamine. L-DOPA and dopamine have the ability to oxidatively polymerize to produce dark polymeric pigments called melanin through a series of enzymatic and non-enzymatic reactions. In mammals, there are two main types of melanin: eumelanin and pheomelanin [28]. The majority of natural melanins are characterized by large particle size, irregular shape, and insolubility in numerous liquids [46, 47]. Some natural melanins exist as small spherical particles, and MNPs can be obtained by high-speed centrifugation (18 000 r/min), for example, those obtained from squid ink sacs by Li et al*.* [48] and Dong et al*.* [49] MNPs with a particle size of less than 10 nm were obtained, or nanoparticles were prepared using nano-preparation techniques such as supercritical carbon dioxide technology, high-density gas technology, and the subcritical water method, among others. The use of modified PEG or liposomes can increase the water solubility of natural MNPs and broaden their applications. Due to its wide availability and biosafety, natural melanin has a high research and application value. The key to the current application of natural MNPs is resolving the issues of particle size, water solubility, and dispersion stability.

## Properties of melanin

### Physicochemical properties of MNPs

The properties of MNPs have the advantages of conventional nanoparticles, such as small size and resistance to phagocytosis. Furthermore, Liu et al*.* suggest that MNPs can passively accumulate in tumors, are not easily recognized and cleared by phagocytes, and can circulate in the body for an extended period [50]. Moreover, the safety, biocompatibility, and biodegradability of MNPs have garnered considerable interest. Liopo et al*.* determined that the cytotoxicity of gold nanorods was double that of synthetic MNPs [51], whereas Pyo et al*.* demonstrated that synthetic MNPs were not cytotoxic at concentrations up to 100 μg/ml [52]. Liu et al*.* discovered that hydrogen peroxide decreased the absorbance of MNPs in solution, demonstrating MNPs' biodegradability [50]. Polyethylene glycolization greatly improved the water solubility and biocompatibility of MNPs, as demonstrated by Liopo. Ju et al*.*, MNPs can scavenge DPPH radicals [53]. Saini et al*.* discovered that biosynthetic MNPs have high radical scavenging rates and electrocatalytic activity, indicating that biosynthetic MNPs have the potential to be used as natural antioxidants and electrochemical biosensors [50]. MNPs can be utilized as photoacoustic contrast agents, as demonstrated by Liopo et al*.* [51] and Zhang et al*.* [54]. Wang et al*.* demonstrated that MNPs were capable of photothermal conversion after their water solubility was enhanced with methoxypolyethylene glycol amine (mPEG-NH_2_) [55]. MNPs can serve as anchoring sites for amine- or thiol-functionalized molecules and are modifiable via Schiff base or Michael addition reactions. Liu et al*.* suggested that thiol-containing and amino-terminated molecules could also be used to modify the surface of MNPs [50]. In addition, MNPs can be loaded with a variety of target groups, and Longo et al*.* demonstrated that the presence of amine groups on the surface of MNPs allows for the conjugation of suitable molecules for in vivo specific targeting [56]. By cross-linking with MNPs, the RGD peptide (cyclic Arg-Gly-Asp-D-phe-Cys [c(RGDfC)]) was successfully targeted to the v3 integral protein overexpressed on the surface of tumor cells.

### Effect of melanin on cancer development

Due to its broad absorption spectrum, natural melanin is traditionally believed to be an effective shield against UV damage, including UVR-induced carcinogenesis and melanoma formation. Recent academic research has demonstrated that melanoma, melanin, and melanogenesis have a yin-yang relationship, i.e., melanin is protective under physiological conditions and destructive under pathological conditions. Melanin may prevent the development of skin cancer, but it may be necessary for the transformation of melanocytes into malignant cells. Melanogenesis and its highly active intermediates exhibit cytotoxic, genotoxic, and mutagenic properties that stimulate glycolysis and hypoxia-inducible factor 1-alpha (HIF-1) activation, promote immunosuppression of tumors, melanoma progression, and immunotherapy resistance [57]. Simultaneously, melanin monomers can generate a pro-oxidant environment and induce DNA damage under unique conditions, creating a mutagenic environment that promotes melanoma formation [58–62]. The yin-yang effect on melanogenesis suggests that the provision of exogenous melanin by MNPs, which circumvents the melanogenic process and the production of intermediates, effectively diminishes the role of melanin in promoting tumor progression.

## Surface modification strategy based on MNPs

Nanomaterial surface modification is a valuable method for enhancing the surface properties of nanoparticles in order to target disease states, increase circulation, and provide targeted payload release in drug delivery. Currently, surface grafting [63], layer-by-layer self-assembly [64], self-assembled monolayer formation [65], Langmuir–Blodgett deposition [66], and plasma [67] treatment are the most prevalent techniques for surface modification of materials. However, they have low chemical specificity, limiting their applicability to various polymer. And although these techniques can achieve multifunctionality of the material surface, they are typically hampered by tedious reaction processes, harsh reaction conditions, and the possibility of altering the material's property. Thankfully, the emergence of melanin films especially synthetic PDA films as a transition layer to modify the surface of materials has substantially compensated for these deficiencies [68]. Moreover, melanin coatings can be used as a secondary reaction platform to adhere functional components such as long-chain molecules, biomolecules, and metal films to their surfaces to form functional coatings. Due to these benefits, the application of melanin coatings in the field of surface modification has become an important research direction and is widely used in biomedical, energy, and industrial settings [69–71]. As PDA-based surface modification is the most widely studied among MNPs, this subsection focuses on PDA-based surface modification strategies.

PDA comprise hydroxyl, carboxyl, phenolic, and amine groups, which offer numerous potential binding or biosorption sites for metal ions to be deposited on the surface of virtually any substance. They are able to graft drugs, bioactive molecules, and polymers onto its surface via addition reactions [72], photoinitiated polymerization [73], free radical polymerization [73], single-electron transfer activated radical polymerization (SET-LPR) [74], surface-initiated atom transfer radical polymerization (SI-ATRP) [75], and reversible addition-rupture chain transfer polymerization (RAFT) [76], thereby enhancing the dispersibility and biocompatibility of PDA or imparting them with slow or controlled release properties.

Addition reactions are a common method for modifying the surface of PDAs, and PDA films can be immobilized with amino- and thiol-containing compounds via Michael addition reactions or Schiff base reactions. For instance, -SH or -NH_2_ capped polyethylene glycols (PEG-SH or PEG-NH_2_) are immobilized on the surface of PDA films by Michael addition reactions, and nanodrugs modified by this step decrease the recognition and destruction of nanoparticles by the reticuloendothelial system (RES) and increase circulation time [55, 77–80]. In addition, using this method, various peptide or ligand molecules can be functionalized onto the surface of PDA-based nanoparticles to enhance tumor cell uptake. Other polymerization strategies can be combined with chemistry to modify the surface of materials. SI-ATRP is one of the most prevalent strategies for obtaining material surfaces with different properties. By combining SI-ATRP with mussel-inspired chemistry, Huang et al*.* first produced a multifunctional polymer coating by dipping SiO_2_ in an aqueous dopamine solution, and then -bromoisobutyryl bromide reacted with the hydroxyl and formed a hydroxyl-functionalized poly(ethylene glycol). Following the amidation reaction of α-bromoisobutyryl bromide with the hydroxyl and amino groups on the surface of the PDA coating, SiO_2_ with bromine groups were obtained. SI-ATRP was then used to graft the polymer onto the surface of the nanomaterials in a solution of water and methanol. By contrast, the surface-modified nanomaterials exhibited higher adsorption efficiency for the azo fluorescent dye Congo Red (CR) and owing to this property, this strategy is anticipated to be utilized in the fabrication of numerous other functionalized polymer nanocomposites for environmental applications [81]. SET-LRP is a recently developed controlled/reactive radical polymerization [82]. It permits the synthesis of polymers with controlled polymer properties, even at room temperature, under mild conditions. And due to the outer SET mechanism's low activation energy, SET-LRP polymerizes rather rapidly. Similar to SI-ATRP, graphene oxide, silica nanoparticles, and carbon nanotubes can be surface modified by introducing amino and hydroxyl groups on their surfaces via the self-polymerization of dopamine and then using these functional groups to immobilize the initiator and graft the polymer onto the surface of the material via SET-LRP [83–87]. In addition to controlled radical polymerization methods, a number of other radical polymerization strategies are used for modification of materials and surfaces [88–92].

## MNPs in oncology treatment

### Imaging

Nanomaterials derived from melanin have potential as biocontrast agents due to the following properties: (1) With broad optical absorption, MNPs can be utilized directly for in vivo PAI; (2) by adsorbing Fe^2+^, Fe^3+^, Mn^2+^, Gd^3+^, etc., melanin-based nanomaterials can be used for MRI; (3) by adsorbing radionuclides such as ^64^Cu, melanin-based nanomaterials can be used for PET; (4) nanomaterials based on PDA can be used for PET; (4) by the integration of different imaging modalities into a single nanoplatform, multifunctional melanin-based biological probes can be constructed and used for multimodal molecular imaging.

#### Photoacoustic imaging (PAI)

In recent years, PAI has emerged as a new non-destructive biomedical imaging technique. When a pulsed laser irradiates biological tissue, the absorber in the tissue absorbs the energy of the photons, resulting in a transient local temperature increase, thermal expansion, and the production of photoacoustic signals. Certain imaging algorithms can reconstruct the photoacoustic signal to create an image of the tissue structure [93, 94]. The intensity of the photoacoustic signals is proportional to the wavelength of light absorbed. Melanin is suitable for photoacoustic imaging because of its strong broadband optical absorption that extends into the NIR region, and Liopo et al*.* demonstrated that the solubility of melanin determines its photoacoustic efficiency [51].

Although MNPs have considerable potential for PAI, they are endogenous chromophores, their sensitivity for PAI is limited by their monotonically broad NIR absorption. In order to overcome this obstacle, Ju et al*.* added readily hydrolyzable citraconamides to the surface of bare MNPs, allowing them to aggregate under weakly acidic conditions within tumors. As the solubility of the pigment determines its photoacoustic efficiency, the physical aggregation of MNPs increases the PA signal in the NIR window of biological tissues due to overlapping thermal fields [95]. These PH-MNPs expand the use of MNPs as an alternative to biological nanoprobes.

**Fig. 2:**
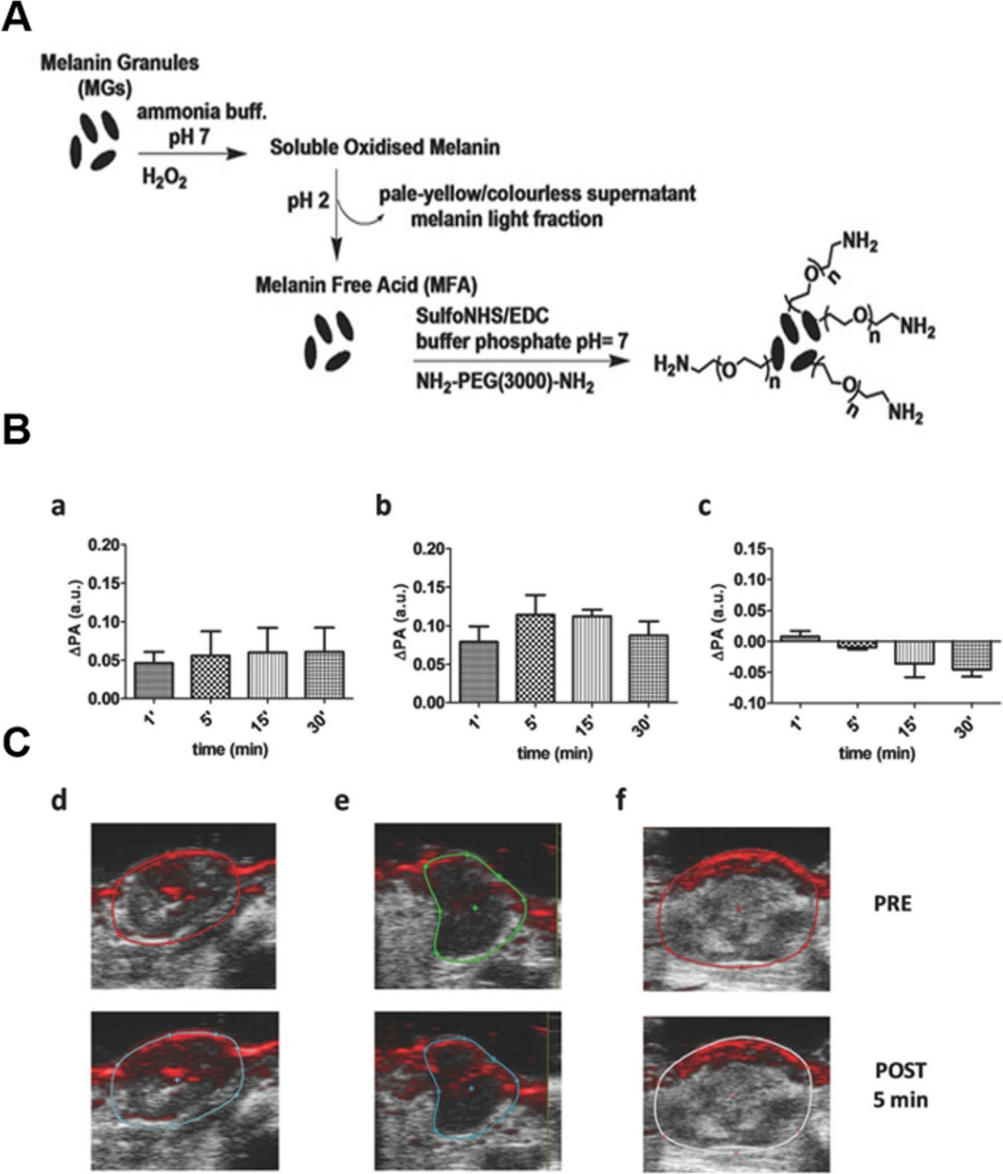
**A** Schematic representation of the synthesis of MFA NPs and MFA-PEG NPs. **B** Mean PA signal changes in mice bearing mammary tumors (n = 4) after intravenous administration of 0.1 mL of a) MFA and b) MFA-PEG or c) 0.1 mL PBS solution at 700 nm at a concentration of 2.5 mg/mL.^−1^.) **C** Representative photoacoustic images of tumor cross-sections before (top) and after 5 min (bottom) of intravenous administration of d) MFA, e) MFA-PEG and f) PBS at 700 nm. Republished from Ref. [56] under the terms of the Creative Commons Attribution Licence (CC BY) (http://creativecommons.org/licences/by/4.0/)

The solubility of melanin determines its photoacoustic efficiency, and high PA contrast is achieved by controlling its aggregation state. Longo et al*.* prepared synthetic melanin particles (SMGs) as a highly water-soluble melanin-free acid (MFA) using a previously reported "bleaching procedure" (Fig. 2) [56]. Nanoparticles of MFA were stored in water for three months without significant size change. The sensitivity of MFA-PEG NPs was examined in vitro at concentrations ranging from 0.6 to 2.5 mg/ml MFA, and the PA signal increased as concentrations of both increased. In addition, MFA-PEG NPs have greater photoacoustic capability as a result of the enhanced transfer efficiency between NPs and water brought about by the PEG coating. In the middle of an HER-positive TS/A tumor model, in vivo tumor imaging capability was evaluated. The PA signal in the tumors of mice treated with both NPs increased gradually over the first 30 min due to the EPR effect, and MFA-PEG had higher contrast photoacoustic properties.

Combining materials with high photoacoustic contrast is another strategy to address the low photoacoustic sensitivity of MNPs. Liu et al*.* loaded the FDA-approved photosensitizer indocyanine green (ICG) into a layered structure such as laponite (LAP), coated PDA onto the ICG-LAP surface, and then used conjugated PEG-RGD as the targeting agent. The resulting ICG/LAP-PDA-PEG-RGD NPs have enhanced ph.

otothermal and photodynamic therapeutic effects, making them a perfect starting point for further integration of nanoplatforms with chemotherapy and PAI [96]. The in vivo PAI results demonstrate the potential of the ICG/LAP-PDA-PEG-RGD nanoplatform as an efficient PA imaging contrast agent.

#### Magnetic resonance imaging (MRI)

MRI is a commonly used diagnostic imaging technique for tumors and has the advantages of high spatial resolution, high tissue resolution, no restrictions on the depth of penetration, and the absence of radiation, but its sensitivity is poor. Gadolinium-based contrast agents (GBCAs) are frequently used clinically to enhance the contrast between tumor tissue and normal tissue [97–99], but they pose a risk of causing nephrogenic systemic fibrosis and gadolinium deposition in brain tissue. Most studies to date have concentrated on T2-weighted MR contrast agents [100, 101]. Generally, T2-weighted magnetic nanoparticles have a low signal-to-noise ratio. This is due to the presence of endogenous negative contrasts in vivo, such as calcium deposition, bleeding, or other metals. By contrast, T1-weighted MR contrast agents can increase their longitudinal relaxation, thereby mitigating the target signal. Chelating Gd ^3+^ or Mn^2+^ ions typically requires an external chelator, such as DTPA or DOTA, for T1-weighted MRI contrast agents [101–103]. Imaging still necessitates high concentrations of panning metals due to the relatively low sensitivity of MRI. Melanin's abundance of functional groups such as amines, carboxyl groups, o-quinones, and semiquinones enables it to chelate with numerous metal ions. Without the need for external chelators, MNPs have been widely used as a building block to chelate different paramagnetic metal ions as MR contrast agents. Typically, precursors such as tyrosine and dopamine are synthesized by enzymatic or chemical polymerization under alkaline conditions [50, 104], after which metal ions such as Mn^2+^, Fe^3+^, Cu^2+^, etc. are anchored to the surface of the nanoparticles, i.e., a post-polymerization doping strategy based on T1 of protons in water, indicating their potential as effective contrast agents for MRI. Ge et al*.* loaded Cu^2+^ onto PDA NPs via a coordination bond and observed that CuPDA NPs were able to shorten the T1 of protons in water, signaling their potential to be an effective contrast agent in MRI [105]. Intriguingly, the longitudinal relaxation rate (*r1*) of CuPDA NPs is superior to that of commercially available Gd-based contrast agents, and in vivo and in vitro experiments demonstrate that these Cu PDA NPs nanoprobes possess enhanced MR contrast.

Li et al*.* used PDA to modify Fe_3_O_4_ to produce Fe_3_O_4_@PDA. Transverse relaxation rates as high as 337.8 mM^−1^ s^−1^ make these Fe_3_O_4_@PDA nanocomposites a promising T2-weighted MRI contrast agent for cancer diagnosis and image-guided cancer therapy [106]. Li et al*.* prepared nanoparticles for MRI/PA dual-mode imaging based on shrinkable gelatin nanoparticles (GNPs) and ultra-small size with Mn^2+^-modified MNPs [107]. Despite the great potential of melanin as an MRI contrast agent, most melanin-like nanoparticles are limited by their large size and poor solubility; therefore, it is important to develop melanin-like contrast agents with excellent solubility and high stability for future clinical application.

**Fig. 3:**
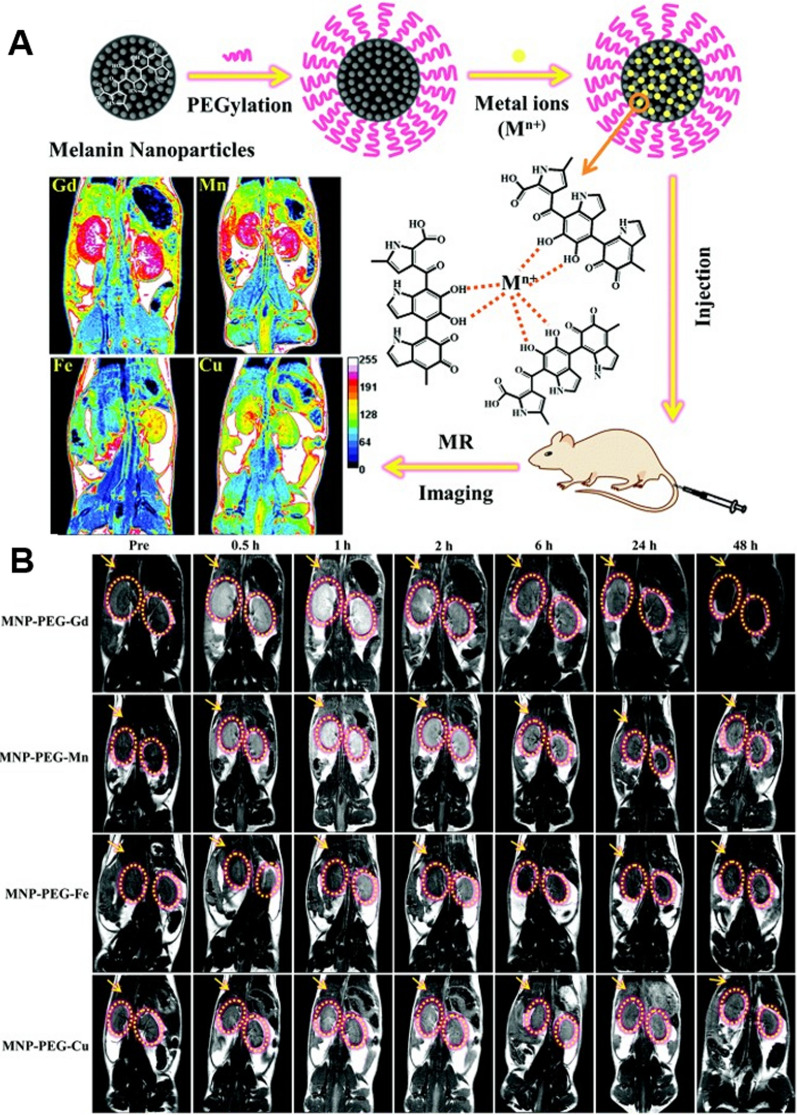
**A** Schematic illustration of the synthesis of metal ion-loaded MNPs for MRI contrast agents. **B** Representative T1-weighted MR images of rats at different time intervals of 0, 1, 2, 6, 24 and 48 h after intravenous administration of different metal ion-loaded MNPs. Republished from Ref. [108] under the terms of the Creative Commons Attribution Licence (CC BY) (http://creativecommons.org/licences/by/4.0/)

Chen et al*.* grafted ultra-small NH_2_-PEG_5000_-NH_2_ onto the surface of water-soluble MNP melanin nanoparticles to create ultra-small NH_2_-PEG_5000_-NH_2_ nanoparticles (Fig. 3) [108]. In addition, the binding properties of melanin to various metal cations (Gd ^3+^, Mn ^2+^, Fe^3+^, and Cu^2+^) were analyzed, and their effects on contrast enhancement under various conditions were compared. The MNP-PEG-M obtained was water-soluble and resistant to biological contamination and aggregation under physiological conditions. ICP-MS was used to detect the concentration of metal ions. It was discovered that the metal ion binding capacity steadily increased with the loading mass ratio of metal ions to melanin. The maximum binding capacity of various metal ions to MNP-PEG was Fe ^3+^  > Cu ^2+^  > Mn ^2+^  > Gd ^3+^, with the difference between their binding capacities possibly being related to the radius of metal ions and ligand geometry. When the mass ratio is 1, the loading of various metal ions reaches the binding saturation point, and excess metal ions can be removed by centrifugation without the precipitation phenomenon resulting from direct chelation of excess metal ions by unmodified MNP on the surface. The binding capacity and relaxation were investigated by varying the mass ratio, with a fixed mass ratio yielding the best in vitro MRI performance. In vivo MRI results demonstrated that the different metal ion chelated melanin nanoparticles exhibited significant MRI signal enhancement and were efficiently excreted via the renal and hepatobiliary pathways, thereby reducing the potentially toxic effects due to long-term accumulation while maintaining high chelation stability and biosafety.

#### Positron emission computed tomography (PET)

Positron emission PET utilizes positron-emitting nuclide-labeled drugs for imaging, which can reflect the metabolic and functional status of focal tissue at the molecular level with good sensitivity, resolution, and safety, and it can play a crucial role in the early detection of tumor metastases. It is essential for the early detection of cancer metastases. Radionuclides (such as ^99 m^ Tc, ^131^I, ^64^Cu^2+^, and ^89^Zr) can be ligated to MNPs, enabling the use of surface-modified MNPs nanoplatforms for both PET and radiotherapy (RT). For example, ^64^Cu^2+^ was chelated onto the surface of Me MNPs by Hong et al*.* for PET to monitor biodistribution and treatment response for guiding treatment [109]. In vivo PET revealed elevated radioactivity concentrations in the livers of mice. Simone et al*.* observed endothelial targeting of PVPh-NP based on PET imaging with radioisotope ^124^I [110]; Yang et al*.* utilized DOX-conjugated, cRGD-functionalized, and ^64^Cu-labeled superparamagnetic iron oxide nanoparticles for PET/MRI imaging and targeted anticancer drug delivery [111].

**Fig. 4:**
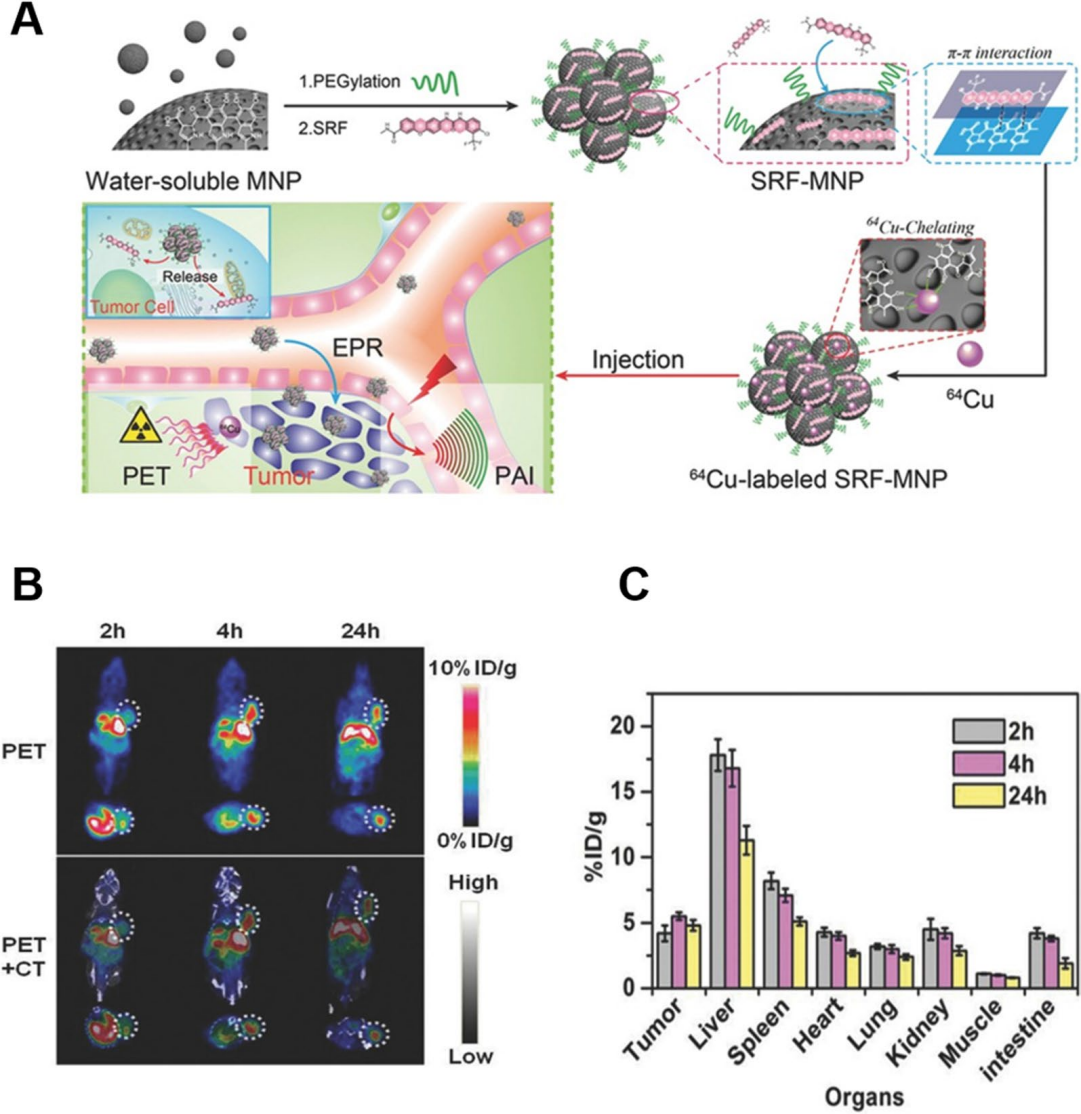
**A** Schematic illustration of the synthesis of SRF-MNP. **B** Representative decay-corrected coronal (top) and transaxial (bottom) small-animal PET images (upper layer) and overlaid CT (grey) and PET (color) images (bottom layer) of HepG2 tumor (region enveloped by white dotted line). **C** Biodistribution of.^64^Cu-radiolabeled SRF-MNP in mice n = 3). Republished from Ref. [112] under the terms of the Creative Commons Attribution Licence (CC BY) (http://creativecommons.org/licences/by/4.0/)

Through EPR effects, radionuclide-functionalized MNPs alter the pharmacokinetics of the drug and provide imaging signals. Zhang et al*.* conjugated sorafenib (SRF) to MNPs via π-π interactions and then chelated radioactive ^64^Cu^2+^ to enable monitoring of the MNP-SRF drug delivery system (Fig. 4) [112]. ^64^Cu^2+^ was labeled at up to 64% by PET observation of biodistribution in mice bearing HepG2 tumors at 24 h post-injection revealed that SRF-MNP in tumors was detected in abundance in the liver (11.3 ± 1.1% ID g^−1^) and spleen (5.1 ± 0.3% ID g^−1^) with ^64^Cu^2+^ radioactivity almost absent in other major organs, indicating that SRF-MNP is primarily cleared through the hepatobiliary system clearance. As SRF is gradually released from MNPs, the drug's biodistribution is comparable to that of MNPs labeled with ^64^Cu^2+^. In addition, MNPs are simpler to prepare, have no potential toxicity, and are better suited as natural platforms than conventional imaging-guided therapeutic nanoplatforms.

#### Multi-modal imaging

**Fig. 5:**
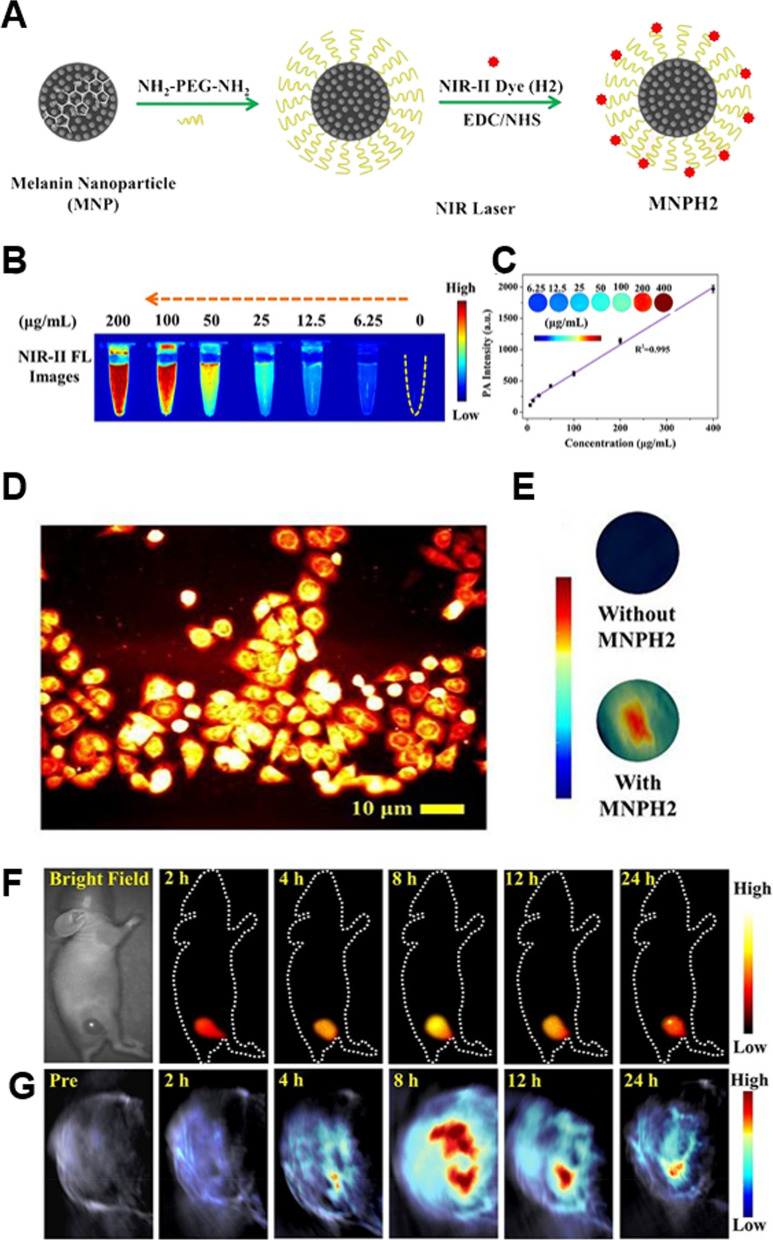
**A** Schematic diagram of MNPH2 synthesis. **B** NIR-II fluorescence images of MNPH2. **C** Linear relationship between concentration and PA signal. Inset: PA images of different concentrations of MNPH2. **D** NIR-II fluorescence images of MNPH2 (100 μg/ml) treated Hep-2 cells. Scale bar = 10 μm. **E** PA images of Hep-2 cells without and with MNPH2 (100 μg/ml) treatment. In vivo imaging: NIR-II fluorescence images of Hep-2 mice treated with MNPH2 at predetermined time points within 24 h. NIR-II fluorescent images **F** and PA images **G**. Republished from Ref. [116]. under the terms of the Creative Commons Attribution Licence (CC BY) (http://creativecommons.org/licences/by/4.0/)

PAI provides deeper optical imaging but is limited by tissue penetration [113]; MRI clearly displays the anatomical structure and boundaries of local lesions but does not provide molecular imaging data [114]; PET imaging is highly sensitive and can detect systemic lesions but lacks spatial resolution [115]. By combining the synergistic advantages of several imaging modalities, multimodal imaging gives complementary diagnostic information. Conventional multimodal nanoprobes necessitate intricate and lengthy synthesis procedures to incorporate multiple imaging contrast components onto one nanoplatform, frequently introducing toxic reagents and raising potential safety concerns that impede the development of additional clinical applications. Melanin-based multimodal imaging probes are desirable because of their natural origin and excellent reactivity. Sun et al*.* developed a novel laryngeal cancer PTT nanoprobe MNPH2 for NIR-II fluorescence/P by combining melanin nanoparticles with the small molecule hydrophobic dye H2 in a single-step EDC/NHS method to enhance the solubility and biocompatibility of H2 and MNPH2. Visible and NIR-II fluorescence spectra of MNPH2 measured in vitro revealed that the NIR-II fluorescence emission spectrum of the MNPH2-sensitive system peaked at 1006 nm and extended well into the NIR-II region when stimulated at 790 nm (Fig. 5) [116]. The NIR-II fluorescence characteristics of MNPH2 were investigated. At 760 nm excitation, PA signals were obtained for varying concentrations of MNPH2 solutions, and it was discovered that the signal intensity increased proportionally with increasing concentration. In vivo investigations assessed the biodistribution and tumor accumulation of the nanoprobe by intravenous delivery of MNPH2 to mice containing Hep-2 cells, utilizing dynamic NIR-II fluorescence/PA bimodal imaging, and discovered that tumors could be identified with ultra-high sensitivity and spatial resolution. Combining melanin nanoparticles with NIR fluorescent dyes is very useful for expanding the therapeutic applications of MNPs and boosting their potential translation into accurate and individualized cancer treatments. Based on its remarkable NIR-II fluorescence and photoacoustic properties, MNPH2 is a remarkable NIR-II fluorescent/PA bimodal contrast agent.

**Fig. 6:**
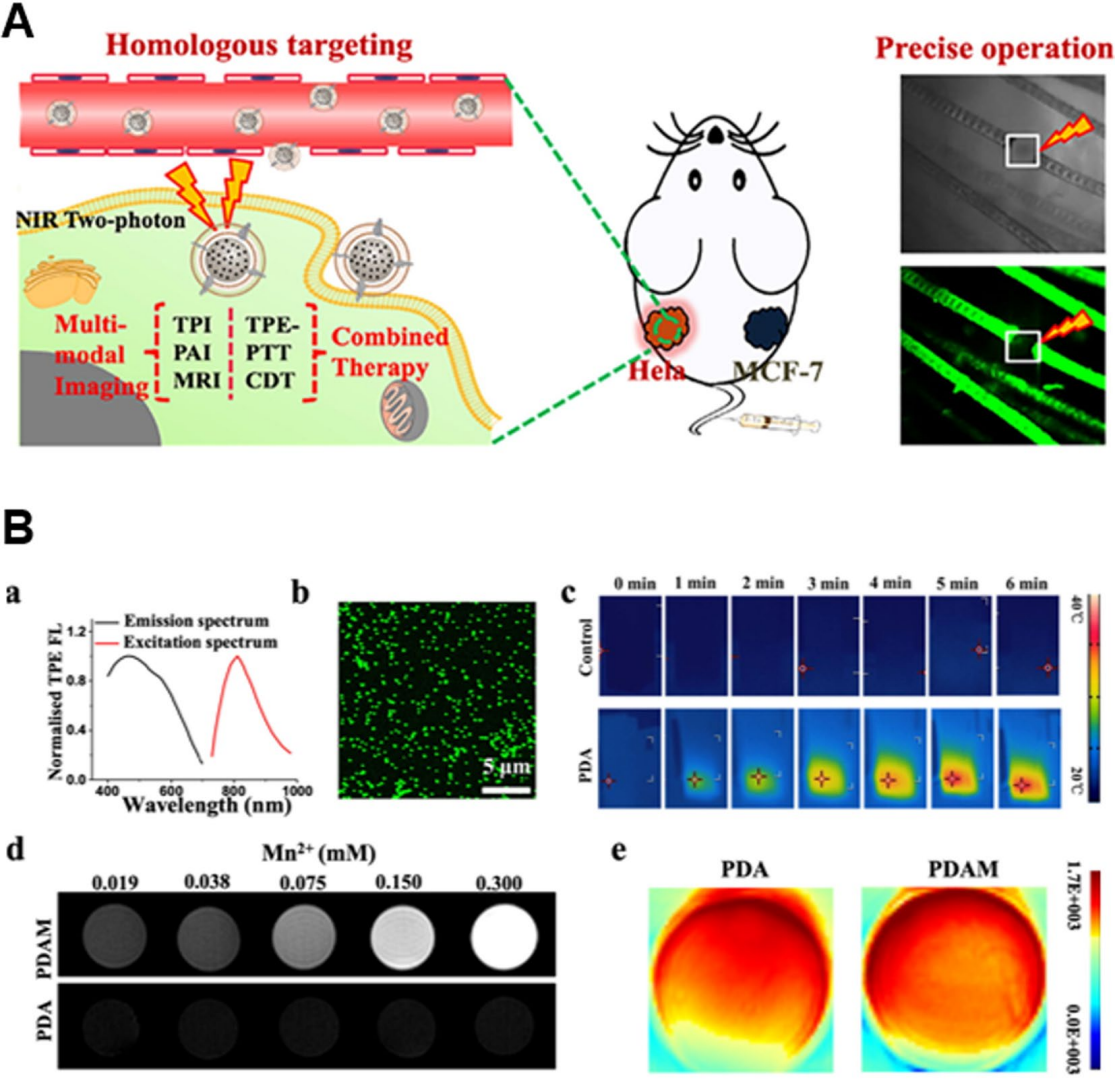
**A** Schematic diagram of PDA &PDAM. **B** Imaging properties of PDA and PDAM. a) Normalized two-photon b) TPE fluorescence. c) Photothermal images of PDA nanoparticles irradiated by two-photon laser for 6 min. d) T1 weighted MR image of PDAM with various Mn2 + concentrations. e) PA images of PDA and PDAM nanoparticles at a concentration of 0.5 mg/ml. Republished from Ref. [117] under the terms of the Creative Commons Attribution Licence (CC BY) (http://creativecommons.org/licences/by/4.0/)

Cao et al*.* considered the phenylethylamine or DHI structure generated during the PDA assembly process with D-π-D bonds, and the conjugation of different compounds increases the length of the conjugate and enhances the coplanar form of the molecule, in accordance with the nature of two-photon materials, to realize the two-photon excitation property of PDA NPs to achieve higher spatial resolution and signal-to-noise ratio images obtained (Fig. 6) [117]. The PDA nanoplatform was also loaded with Mn^2+^ -PDAM, which endowed the PDA NPs with MRI capabilities. The in vitro and in vivo results showed that PDA exhibited weak fluorescence and single-photon absorption, but had strong two-photon nonlinear optical processes. In addition, PDAM loaded with Mn2 + has both NIR two-photon absorption and MRI capabilities, allowing the material to act as a multimodal bioimaging agent.

Although nanoparticles based on MNPs, especially PDA nanoplatforms for tumor imaging, have been widely studied, PDA itself has poor imaging properties [95, 117]. To solve this problem, it is.

necessary to improve its solubility or graft other substances with imaging capabilities on the nanoplatform, which leads to more complex preparation processes and pharmacokinetic studies, limiting its clinical research, translation and application.

### Oncology treatment

Nanomaterials derived from melanin can offer a variety of noninvasive strategies for disease treatment: (1) Melanin has an exceptional drug loading capacity and ion chelating ability, and it can transport drugs for sustained and controlled drug release. (2) melanin has a high photothermal conversion efficiency (PCE), making it an ideal photothermal agent for PTT; (3) melanin can also be used for immunotherapy as local heat can activate inflammatory cytokines in the immune system; (4) MNPs and PDA can be used as a biocompatible nanocarrier to transport drugs and photosensitizers (PS) to tumor tissue for cancer chemotherapy (CT) and photodynamic therapy (PDT); (5) synergistic treatment with MNPs can be achieved by combining different therapeutic approaches.

#### Chemotherapy (CT)

Chemotherapy is one of the most prevalent oncological treatments [2, 3]. As chemotherapy is a systemic treatment, however, the selectivity of chemotherapeutic drugs is low, and therapeutic effects are frequently accompanied by varying degrees of toxic side effects [118–121]. Many chemotherapeutic agents, including cisplatin [122], doxorubicin (DOX) [123], irinotecan [124], and pentafluorouracil [125], are enabled by mussel-inspired chemistry to form nanoparticles with sustained controlled drug release and the desired pharmacokinetic properties based on the adhesive and highly reactive nature of polydopamine. In a recent study, Zhang et al*.* encapsulated DOX in mesoporous ZIF-90 using a one-pot synthesis and coated PDA on the ZIF-90@DOX surface using a hard-template technique [123]. The in vitro drug release assay results revealed that the drug's release was pH-dependent. DOX was released at a cumulative rate of 28.19 percent at a PH of 7.4, but this rate increased to 29.94 percent in an acidic environment due to the slow dissolution of PDA. As the microenvironment of tumors, endosomes, and intracellular lysosomes are weakly acidic, this drug-release effect of responsive PH could enhance therapeutic anticancer effects and minimize potential damage to normal cells caused by the acidic tumor microenvironment [126]. This effect also holds great promise for tumor therapy.

**Fig. 7:**
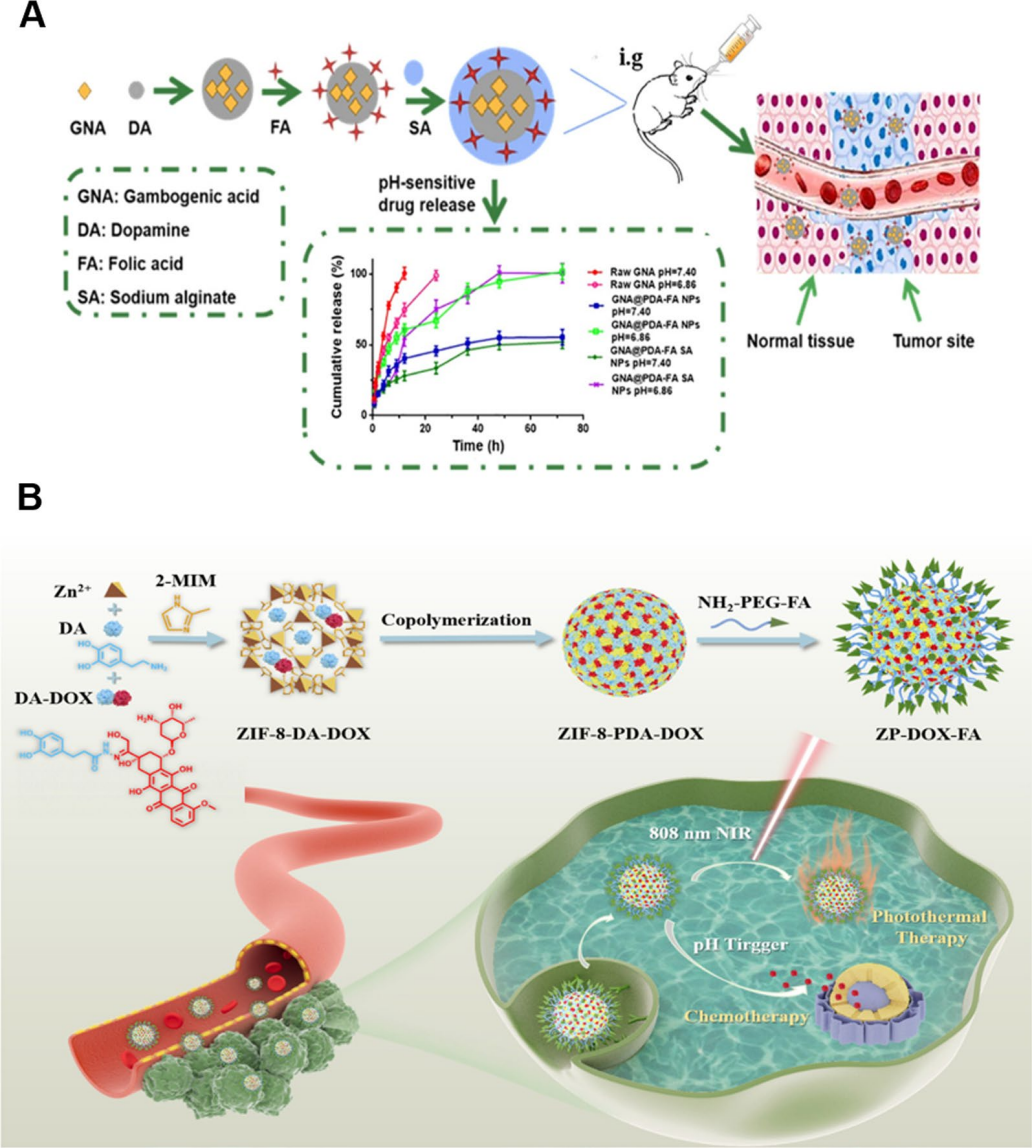
**A** Schematic representation of the preparation of GNA@PDA-FA. Inset: cumulative release profile of GNA released from each NPs (pH = 6.86, 7.40, 37 ± 0.5 °C). **B** Schematic representation of the preparation of ZP-DOX-FA.Republished from Ref. [129, 130]. under the terms of the Creative Commons Attribution Licence (CC BY) (http://creativecommons.org/licences/by/4.0/)

Current conventional chemotherapeutic drugs lack targeting and may be toxic to normal cells while killing cancer cells, resulting in intolerable side effects. Several targeting ligands, including nuclear adaptors (AS1411 and peptides) [127], small molecules (folic acid) [128], and epidermal growth factor (EGFR) [129], can be modified with polyethylene glycol chains (PEG) at the ends and grafted onto the surface of PDA under mild conditions due to the high responsiveness of PDA. (FR), which is overexpressed in many cancer cells, was used as an active targeting ligand in a nanoscale drug delivery system and modified on the surface of PDA-coated gambogenic acid (GNA) nanoparticles via Michael addition reactions **(**Fig. 7). The IC50 of GNA@PDA-FA NPs grafted with folic acid molecules was significantly lower than that of GNA (4.80 M) and GNA@PDA (7.57 M), indicating that the folic acid-modified GNA@PDA-FA NPs were more toxic to tumor cells. The antitumor efficacy of the NPs grafted with FA was confirmed by observing the mean tumor volume, tumor mass, and body weight of mice with 4T1 tumors in vivo.

PDA can be used as a kind of coating, and its coupling with some drugs can only enable co-assembly into nanospheres through van der Waals forces or π-π stacking interactions to improve the pharmacokinetics of the drugs. A pH-sensitive dopamine-derived precursor drug (DA-DOX) was synthesized by Ma et al*.* (Fig. 7) [130]. using an acid-unstable hydrazone linker. Then DA-DOX was encapsulated in the pores of ZIF-8 through π-π stacking interactions. Subsequently, DA-DOX and DA gradually copolymerized in the porous channels of ZIF-8, which simultaneously enhanced the stability and dispersion of ZIF-8 and imparted good photothermal conversion. Then, the tumor-targeting nanocomposite ZP-DOX-FA was obtained by coating the nanoparticles with amino-terminal folic acid-polyethylene glycol (NH_2_-PEG-FA) for subsequent PEGylation.

#### Photothermal therapy (PTT)

**Fig. 8:**
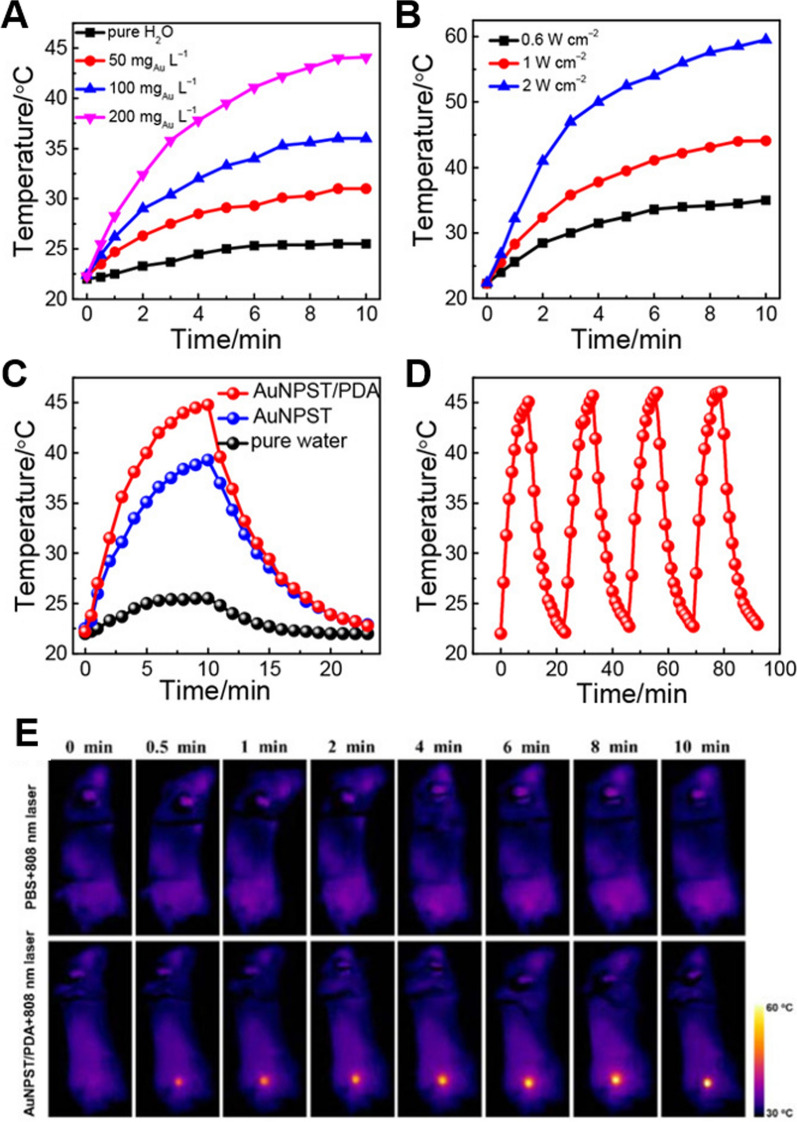
**A** Effect of AuNPST/PDA NPs concentration on temperature increment. **B** Effect of optical density on temperature increment. **C** Temperature change curves of pure H_2_O, AuNPST and AuNPST/PDA aqueous solutions (200 mg Au/L) under 808 nm NIR laser (1 W*cm.^−1^,10 min). **D** Solution temperature variation profile of NP after four on/off cycles of 808 nm NIR laser. **E** In vivo photothermal effect of AuNPST/PDA NPs. Republished from Ref. [144] under the terms of the Creative Commons Attribution Licence (CC BY) (http://creativecommons.org/licences/by/4.0/)

Tumor hypoxia, caused by rapid tumor cell proliferation and limited neovascularization, is a crucial characteristic of the tumor microenvironment. The tumor microenvironment tends to be hypoxic, hyperprotonated, and reductive, prompting tumor cells to adapt to the unfavorable environment via diverse cellular mechanisms, thereby enhancing drug resistance and survival [131–134]. Photothermal therapy (PTT), a therapy involving artificially elevated tissue temperature, has demonstrated superior performance in the field of cancer treatment due to its non-oxygen dependence, lower invasiveness, and high intrinsic specificity [135–137]. Numerous materials, such as carbon nanotubes, chemographene, conjugated polymer nanomaterials, gold nanorods, and PDA nanospheres, have been demonstrated to be capable of photothermal conversion [138–141]. Nevertheless, numerous photothermal converters have evident limitations. Inorganic photothermal converters are cytotoxic [142], and polymeric photothermal converters such as polypyrrole nanoparticles are similarly hard to degrade in vivo [143]. Using site-specific growth techniques, Pan et al*.* coated PDA onto mechanically unstable gold nanoparticle superstructures (AuNPST) without linkers to enhance the efficacy of photothermal cancer therapy (Fig. 8) [144]. The prepared AuNPST/PDA is significantly more heat-, acid-, and base-resistant. Moreover, under 808 nm laser irradiation, AuNPST/PDA demonstrated a higher photothermal conversion rate (33.3 percent) than AuNPST alone (23 percent). Photothermal therapy was validated in vitro by incubating Hela cells with 200 g/ml nanoparticles for six hours. When irradiated at 808 nm, approximately 99 percent of the cells remained viable, and more than 80 percent of the cells were killed, demonstrating the excellent tumor ablation effect of NPs. In vivo photothermal treatment confirmed this outcome. Notably, during the in vivo PTT validation, the major organs (liver, spleen, and kidney) of the AuNPST/PDA mice group were found to be healthy. Images of the major organs (liver, spleen, and kidney) of AuNPST/PDA mice stained with H&E revealed no significant damage, and blood biochemical and hematological analyses revealed no significant toxicity of the PDA-coated NPs, indicating the PDA-coated NPs' increased biocompatibility.

#### Immunotherapy

In recent years, tumor immunotherapy has emerged as an effective anti-tumor method; however, studies have found that the lack of immunotherapy efficacy in solid tumors such as liver cancer can be attributed to the lack of suitable tumor antigens as targets and an inhibitive tumor microenvironment [145–149]. In the microenvironment of solid tumors, hypoxia, nutritional deficiencies, and the expression of inhibitory checkpoint ligands inhibit the proliferation and effector functions of T cells, making it difficult for T cells to enter cancerous tissues to exert anti-tumor effects, and even if they do, their function is impaired, making it difficult to ensure the sustained and effective anti-tumor activity of intratumor T cells. In addition, the conventional method of systemic administration leads to overactive immune cells in the body, which attack normal tissues in the process of killing tumors, resulting in a series of adverse immunotherapeutic reactions, such as cytokine release syndrome and neurotoxicity. It has been reported that nanoparticles with a diameter between 20 and 200 nm have a greater chance of entering capillary lymphatics, reaching lymph nodes, and being ingested by macrophages and dendritic cells [150]. Antigenic peptides with nucleophilic groups can be grafted onto the nanoparticle surface by binding to catechol groups via Michael addition or Schiff base reactions, and the melanin-based nanoplatform is enriched around the tumor site with the nanoparticles and directly captured by antigen-presenting cells (APCs) to initiate a series of tumor immune responses.

**Fig. 9:**
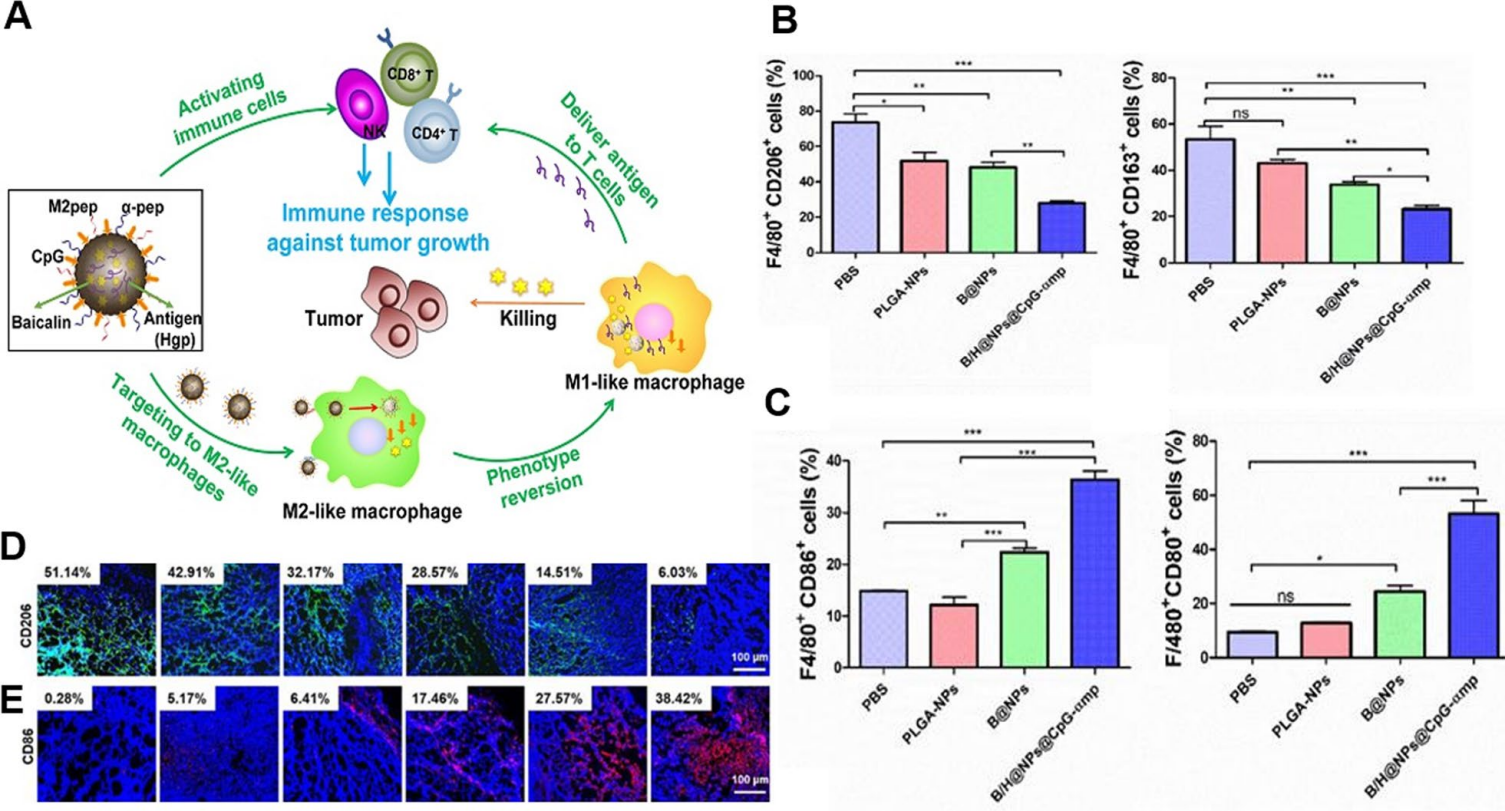
**A** Schematic representation of the role of B/H@NPs@CpG-αmp NPs in targeting to M2-like TAMs and activating immune cells. **B**, **C** Expression of CD206 and CD163 in M2-like TAMs after treatment with different agents. **D**, **E** Immunofluorescence detection of M2-like and M1-like TAMs. blue: nuclei, red: CD86 + cells, green: CD206 + cells. Republished from Ref. [151] under the terms of the Creative Commons Attribution Licence (CC BY) (http://creativecommons.org/licences/by/4.0/)

Han et al*.* encapsulated baicalin and melanoma antigen Hgp peptide fragments 25–33 in poly(lactic acid-glycolic acid) (PLGA) nanoparticles using the ultrasonic double emulsion technique and then coated the nanoparticles with a polydopamine film to stabilize them (Fig. 9) [151]. CpG-ODN modifies the tumor-associated macrophage (TAM) phenotype from M2-like to M1-like. Nanoparticles that dual-target M2 TAMs are enriched in the tumor due to their nanoparticle properties. The dual targeting of M2 TAMs by tumor microenvironment-enriched nanoparticles effectively targets M2-like macrophages and delivers the immunostimulant CpG, further reversing the conversion of M2-like TAMs to M1-like TAMs. M1-like TAMs that are mature and activated serve as a secondary delivery vector by presenting antigens that activate T cells. Simultaneously, M1-like TAMs and activated T cells secrete inflammatory cytokines, including IL-12, TNF-α, to promote necrosis of tumor cells and immune cell infiltration. The release of baicalein encapsulated in PLGA following nanoparticle phagocytosis stimulates immune cells synergistically. Experiments in vitro revealed that the expression of CD163 and CD206 (markers of M2-like TAMs) decreased in macrophages exposed to NPs, while the expression of CD80 and CD86 (markers of M1-like TAMs) increased significantly, indicating the transformation of M2-like TAMs. In vivo TAMs were analyzed after intravenous administration of the nanocomplex, and the same in vitro experiments were supported by comparing the area ratio of cells expressing M1-like biomarkers (CD206 and CD163) to areas expressing M2-like biomarkers (CD86 and CD80) in tumor tissue.

#### Gene therapy

**Fig. 10:**
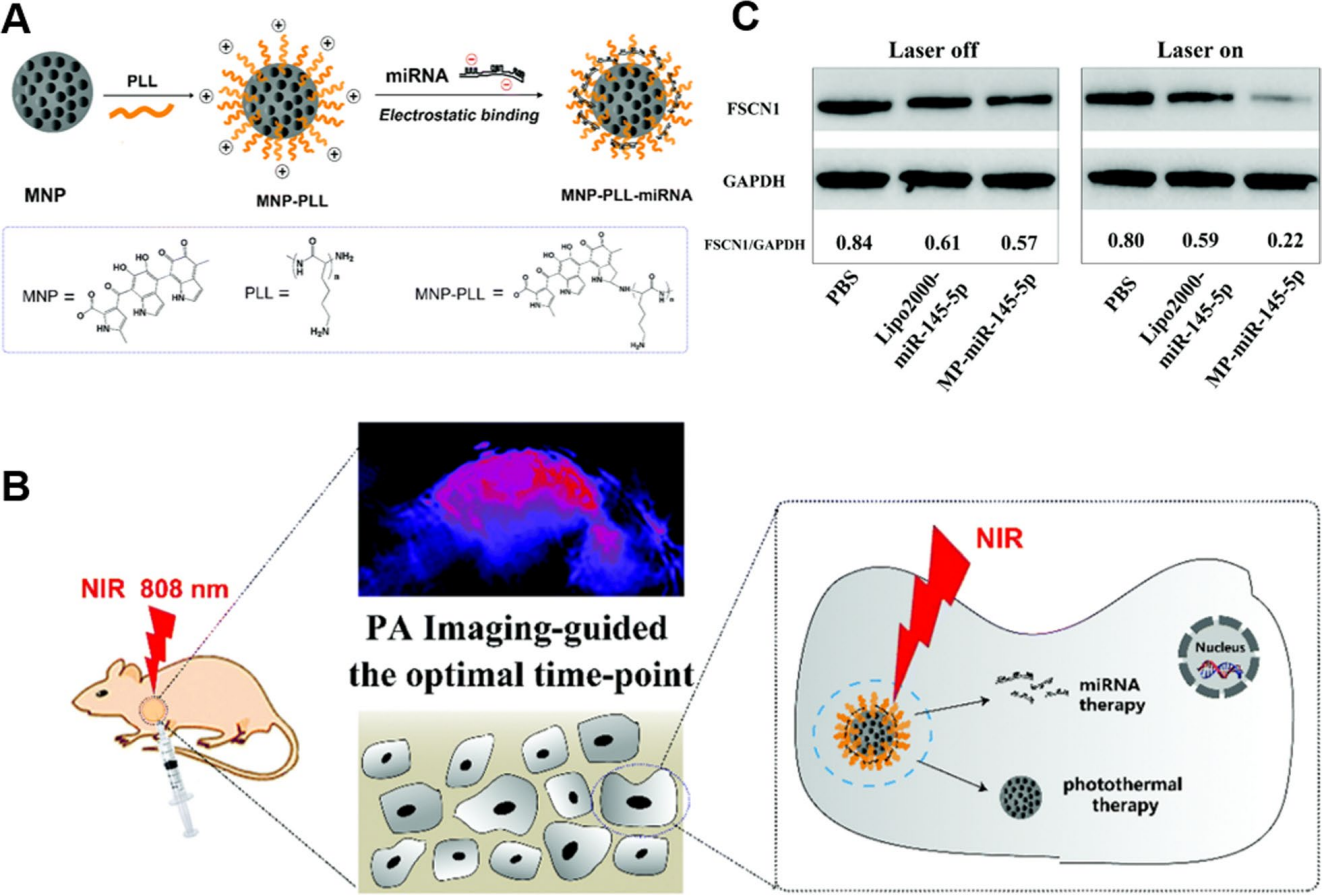
**A** Schematic diagram of the preparation process of MNP-PLL/miRNA NPs. **B** Application of MNP-PLL/miRNA NPs in genePTT. **C** In vitro Western Blotting assay. Republished from Ref. [155] under the terms of the Creative Commons Attribution Licence (CC BY) (http://creativecommons.org/licences/by/4.0/)

**Fig. 11:**
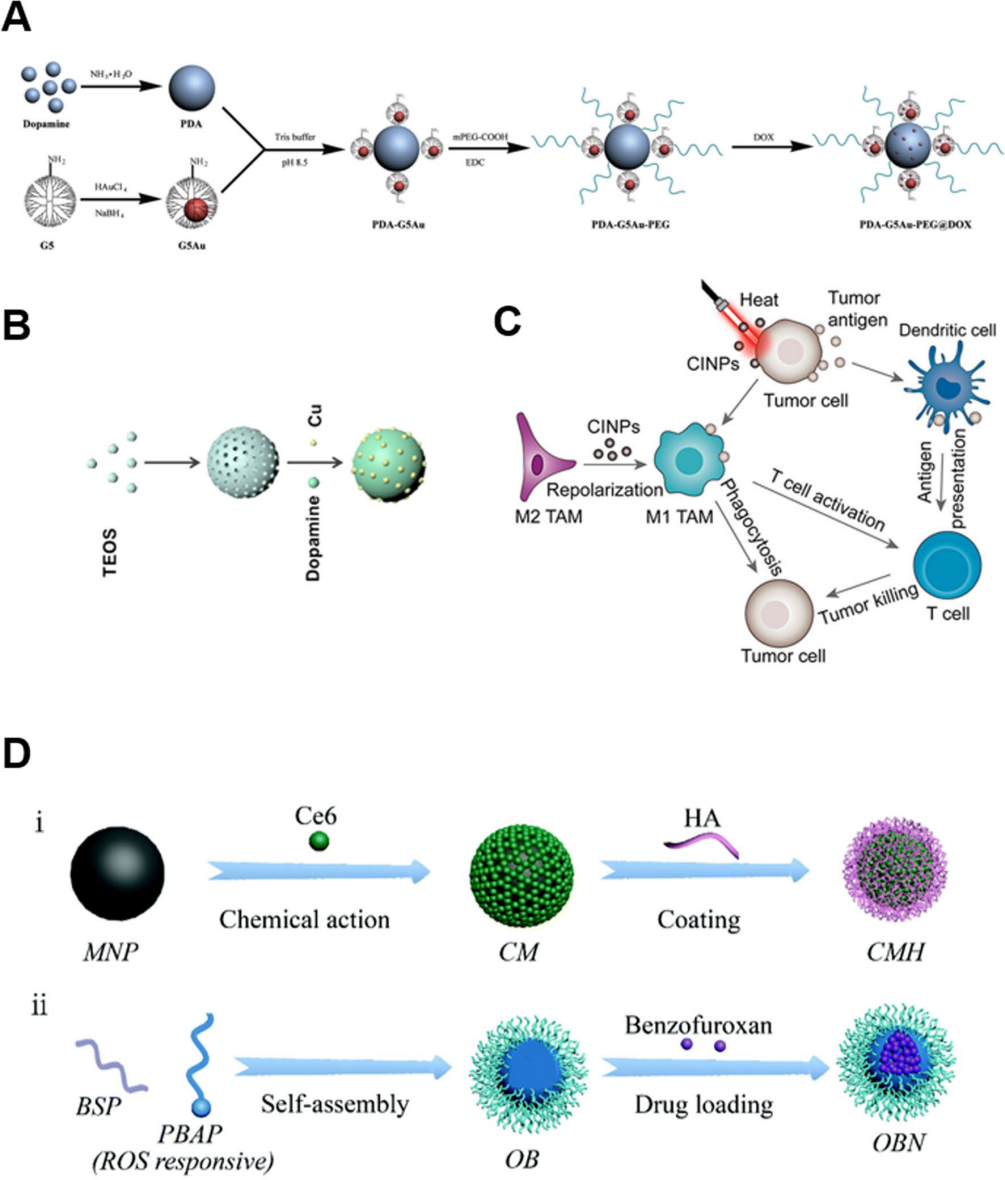
**A** Schematic illustration of the synthesis of PDA-G5Au-PEG@DOX NPs(**A**), HMSNs@PDA-Cu(**B**), CINPs(**C**), MNPs(**D**). Republished from Ref. [157, 158], [163] under the terms of the Creative Commons Attribution Licence (CC BY) (http://creativecommons.org/licences/by/4.0/)

As gene mutations can lead to tumor development, in addition to chemotherapeutic drugs, therapeutic genes such as DNA, miRNA, and siRNA can be transported into target cells via appropriate vectors to precisely up-/down-regulate the expression of specific tumor-related genes and inhibit tumor development, thereby achieving the objective of cancer therapy. As exogenous genes, DNA can be integrated into the recipient's genome after it has been transported into the cell in order to encode specific therapeutic proteins with therapeutic effects [152–154]. However, disadvantages such as susceptibility to nuclease degradation, short circulation time, poor cellular uptake, and low transfection efficiency suggest that delivery of therapeutic genes remains the greatest obstacle to their application. Gene therapy requires the immediate development of safe and effective nanovector systems. Due to the chemical reactivity of melanin-like nanoparticles, positively charged substances such as lysine (PLL) and polyethyleneimine (PEI) can be grafted onto their surface to create positively charged nanoparticles. Electrostatic interactions or chemical bonds can form carrier-gene complexes between positively charged nanocarriers and densely negatively charged nucleic acids. For example, Fan et al*.* loaded PLL onto the surface of melanin nanoparticles after the human cancer anticancer gene Hsa-miR-145-5P coalesced on the surface of the melanin nanocomplex by electrostatic interactions to produce (MNP-PLL/miRNA NPs) (Fig. 10) [155]. Using confocal laser scanning microscopy (CLSM), it was discovered that MNP-PLL/miRNA NPs were more efficiently internalized by Hep2 cells than miPNA alone. Experiments involving protein blotting in vitro revealed diminished expression of the FSCN1 protein, a crucial inhibitor of tumorigenesis and metastasis. miR-145-5p is a negative regulator of FSCN1 expression in laryngeal squamous cell carcinoma (LSCC), and its overexpression decreased FSCN1 expression. In an additional setting, we compared the expression of FSCN1 with and without laser irradiation and found that MP-miR-145-5p induced 40% downregulation of FSCN1 expression in the presence of NIR irradiation compared to in the absence of NIR irradiation, demonstrating that gene regulation could be enhanced by the photothermal effect. In vitro studies of the antitumor effects of NPs revealed that MP-miR-145-5p without NIR laser moderately inhibited tumor progression, whereas MP-miR-145-5p + NIR demonstrated effective tumor suppression, indicating that photothermal therapy may be a key factor in the suppressive effect Fig. 11.

#### Combination therapy strategies including PTT

As previously stated, melanin possesses a number of unique properties, most notably excellent light absorption; therefore, PTT based on nanoplatforms resembling melanin is the most established. However, single PTTs still have limitations, such as limited tissue penetration of light, upregulation of heat shock protein expression resulting in heat resistance of tumor cells, damage to surrounding normal tissue due to heat diffusion, and inadequate release of tumor-associated antigens [156]. Fortunately, due to the ease of surface functionalization by other materials, melanin-based nanoparticles can be used to construct multifunctional platforms for enhancing the overall efficacy of PTT in combination with other therapies.

Chemotherapeutic agents are the predominant treatment in clinical oncology and penetrate tumor tissue more deeply than nanomaterials. Surface-modified nanoplatforms resembling melanin can effectively combine the benefits of CT and PTT [54]. MNPs can bind to drugs in a similar manner and serve as a drug delivery system for dose reduction and side effects. Wang et al*.* [157] designed a nanocarrier with a core-satellite structure, PDA-G5Au-PEG@DOX, using polydopamine (PDA) as the core, 5th generation dendrimer/gold (G5Au) nanoparticles as satellites, and the chemical DOX loaded onto the surface of the core-satellite nanocarrier. When these satellite NPs reach the tumor site, the acidic microenvironment of the tumor triggers the dissociation of PDA, resulting in the release of the drug. Simultaneously, the core PDA is irradiated with NIR to promote tumor cell ablation by PTT, thereby achieving the CT-PTT combined treatment. Experimentally, G5Au is used to enhance PTT and enhance the penetration of NPs into deep tumors. By co-localizing tumor vasculature with DOX, DOX fluorescence was observed away from the vasculature, indicating that NPs could deliver DOX to the internal regions of solid tumors, as opposed to free drugs. In vivo tumor growth inhibition assays demonstrate that PDA-G5Au-PEG@DOX NPs can achieve a synergistic effect between CT and PTT in the treatment of tumors.

Nanoparticles resembling melanin have the potential to combine the benefits of both materials. Zhang et al*.* utilized HMSNs to deliver PDA and copper ions (Cu(II)) [158]. Cu(II) was reduced by intracellular glutathione (GSH), and Cu (I) then underwent a Fenton-like reaction with hydrogen peroxide (H_2_O_2_) to generate hydroxyl radicals (-PDA enables photothermal therapy) (PTT). The investigation also investigated PDA's superoxide dismutase (SOD) mimetic activity to boost H_2_O_2_ production for synergistic CDT with Cu (II). Additionally, copper ions were ligated with PDA to increase their photothermal conversion efficiency.

Melanin-like nanomaterials can deliver tumor antigens, immune adjuvants, cytokines, and the like for immunotherapeutic purposes. However, in tumor patients, the "cancer immune cycle" is not optimal, as evidenced by inadequate or ineffective exposure to tumor cell antigens, which does not promote the maturation of dendritic cells for immune response. Even when the immune system is activated, the activated cytotoxic T cells have difficulty infiltrating or reversing to regulatory T cells due to the tumor microenvironment (e.g., high interstitial pressure, hypoxia, etc.); the infiltrated cytotoxic T cells may misidentify the tumor antigens as non-antigens and reverse to regulatory T cells, and the infiltrated cytotoxic T cells may misidentify the tumor antigens as non-antigen. Tumor cells may also mistake tumor antigenic material for non-antigenic material and revert to regulatory T cells; tumor cells escape through camouflage, which includes multiple aspects such as antigen-presentation deficiency, induction of apoptosis in cytotoxic T cells, and expression of suppressor molecules (e.g., PD-L1, immunosuppressive cytokines VEGF, TGF and IL-10 [159–161]. Moreover, the administration of immunotherapeutic agents alone can result in systemic adverse effects, such as gastrointestinal toxicity and toxic reactions due to systemic immune responses. To increase the useful release of antigens from tumor patients, enhance dendritic cell function, modulate the tumor microenvironment and immune tolerance, and target the administration of immunotherapeutic agents, new paradigms of tumor immunotherapy have been developed. Several studies have demonstrated that photothermal therapy mediated by nanomaterials can facilitate the activation of the immune system by causing cell necrosis and, consequently, the release of antigenic molecules [162]. By activating the MAPK signaling pathway, nanoparticles revert the tumor-associated macrophage (TAM) phenotype from an immunosuppressive M2-like phenotype to an antitumor M1 phenotype. CINPs also exhibit significant photothermal effects. The tumor-associated antigen produced by PTT and the tumor cell fragments that accompanied the repolarized TAM ablation stimulated an efficient immune response.

To achieve complete ablation of tumors, it is frequently necessary to heat the tumor to temperatures above 50 °C using a powerful laser; however, painful procedures and severe damage to healthy tissue limit its practical application. Nitric oxide (NO) is a relatively reactive free radical molecule that plays a vital role in the metabolic processes of living organisms. It can transmit information between and within cells and is essential for cell communication. High concentrations of NO can directly induce cancer cell apoptosis, and combining PTT therapy with NO gas therapy can help circumvent these issues and improve therapeutic efficacy. Ding et al*.* prepared a cascade release nanoplatform of NO/ROS/RNS for gas/PDT/PTT/tumor immunotherapy consisting of Ce6-melanin-hyaluronic acid nanoparticles (Ce6- MNP-HA, CMH) and a natural nanoplatform made up of Oxi-BSP-NO donor (OBN) microcapsules containing a NO donor [163]. Not only does melanin possess exceptional photothermal properties, but it also aids Ce6 in producing large quantities of reactive oxygen species (ROS) in the near-infrared. ROS-sensitive Oxi-BSP-NO microcapsules simultaneously release NO donors stimulated by CMH-generated ROS, which further release NO in the tumor microenvironment's highly expressed GSH, while NO further upregulates the sGC-cGMP signaling pathway to alleviate hypoxia and synergistically enhance PDT. In addition, the cascade of ROS and NO generates more cytotoxic RNS, which initiates apoptosis and activates T cells in order to improve immunotherapy. Thus, it is anticipated that the combination of ROS, NO, and RNS on a single nanoplatform with high biosafety will result in an accurate and integrated anti-tumor strategy.

Although many researchers have designed a variety of multifunctional nanoparticles based on MNPs, especially PDA, we did not find any pharmacokinetic studies on MNPs and PDA in the database. Most of papers in the databases including web of science, ScienceDirect, SpringerLink and Pub Med are mainly about drug design and validation of the efficacy of the designed nanoparticles. In addition, MNPs are commonly used in PTT. However, the in vivo ability of photothermal agents has only been tested in a few animal studies, and few of them have been approved in clinical trials. To enhance the clinical application of PTT, it is important to expand the knowledge about the therapeutic efficacy of PTT nanocarriers Table 1.

**Table 1:** Experimental studies of surface-modified MNPs in tumor therapy

Applications		NPs	Sources	Preparation method	Surface modification agent	Major output	Ref.
Imaging (Single imaging to multimodal imaging)	PAI	pH-MNPs, MFA & MFA-PEF NPs	Dopamine hydrochloride, Synthetic melanin granules (sMGs)	Oxidation and self-polymerization	mPEG & citraconic amide	•Fourfold higher PA signal for bare MNPs compared to gold nanorods at a given weight concentration, while PH-MNPs showed comparable PA signal intensity to equivalent concentrations of gold nanorods •High water solubility with photoacoustic imaging capability •Good in vivo imaging of tumors •PEG coating improves the transfer efficiency between NPs and water and has higher photoacoustic properties	[56, 95]
	MRI	MNP-PEG-M	Melanin	Oxidation and self-polymerization	NH_2_-PEG-NH_2_ and metal ions	•The best in vitro MRI performance can be obtained at a fixed metal ion-to-melanin mass ratio •The various metal ion chelated melanin nanoparticles not only exhibit significant in vivo MRI signal enhancement, but also can be efficiently excreted via renal and hepatobiliary pathways, reducing the potential toxicity due to long-term accumulation	[108]
	Multimodal imaging:NIR-II/PA Fluorescence	MNPH2	Melanin	HCl neutralizes melanin dissolved in NaOH	NH_2_ -PEG_5000_ -NH_2_	•Excellent NIR-II fluorescent/PA bimodal contrast agent • May serve as a favorable photothermal ablative agent for cancer therapy •Simple design combining melanin nanoparticles and NIR fluorescent dye facilitates expansion of its clinical applications	[116]
Oncology Treatment (Monotherapy to combined therapy)	CT	GNA@PDA-FA	DOPA	Oxidation and self-polymerization	FA	•Higher inhibition of 4T1 cells by NPs through ligand-receptor interactions between FA grafted on PDA coating and FR on cell membrane •Longer blood circulation time •Enhanced bioavailability	[129]
	Immunotherapy	B/H@NPs@CpG-αmp	Dopamine hydrochloride	Oxidation and self-polymerization	M2pep & α-pep	•Dual targeting of M2-like tumor macro phages •Remodeling of tumor micro- environment	[151]
	Gene therapy	MNP-PLL/miRNA NPs	Melanin	HCl neutralizes melanin dissolved in NaOH	Polylysine (PLL)	•MNP-PLL/miRNA NPs can be efficiently internalized by Hep2 cells •Upregulation of miR-145-5p upon entry of MNP-PLL/miRNA NPs into cells results in reduced expression of FSCN1 protein, a key inhibitor of tumorigenesis and metastasis	[155]
	PTT/CDT	HMSNs@PDA-Cu	Dopamine hydrochloride	In situ autopolymerization of dopamine	Cu(II)	•PDA enables photothermal therapy (PTT) •PDA has superoxide dismutase (SOD)-mimetic activity, which enhances H_2_O_2_ production to synergize CDT with Cu(II) •Cu(II) ligands with PDA to further enhance the photothermal conversion efficiency of PDA	[157] [158]
	PTT/Immunotherapy	CINPs	Melanin	Extracted from cuttlefish ink	/	•CINPs reverse tumor-associated macrophages (TAM) from an immunosuppressive phenotype M2-like phenotype to an anti-tumor phenotype M1 through activation of mitogen-activated protein kinase (MAPK) signaling pathway •Tumor-associated antigens produced by PTT and tumor cell debris accompanying TAM repolarization ablation triggered an effective immune response	[162]
	gas/PDT/PTT/immunotherapy	Ce6-MNP-HA (CMH), Oxi-BSP-NO	Melanin	Extracted from cuttlefish ink	HA	•Cascade release nanoplatform of NO/ROS/RNS for gas/PDT/PTT/tumor immunotherapy •Melanin not only has excellent photothermal properties, but also helps Ce6 to generate large amounts of reactive oxygen species (ROS) in the near infrared	[163]

## Concluding remarks and perspectives

Due to their easy preparation, high chemical reactivity, and biocompatibility, nanoparticles resembling melanin hold great promise for oncology therapeutics. However, we have not yet located a systematic review of melanin-like nanoparticles in oncology therapy, and the strategies of cutting-edge pharmacology have not received sufficient consideration. In this paper, we aim to review the definition, preparation, and surface modification strategies of MNPs, focusing on recent advances in tumor diagnosis and treatment, particularly on PTT, and to provide a more comprehensive review from a cutting-edge pharmacological perspective. Despite the fact that a large number of studies have demonstrated the efficacy of this therapy in the field of oncological treatment, its clinical translation still faces obstacles.

Initially, the design of nanoplatforms was based on the following: (a) the therapeutic mechanisms of nanoparticles are complex and require further investigation, and the standardization of treatment is even more challenging to define. Targeting strategies can help to improve the therapeutic efficacy of MNPs, while visualization strategies can aid in understanding the therapeutic mechanism and evaluating the therapeutic effect in real-time, thereby promoting their clinical application. Based on MNPs, the development of PTT with photothermal-immunotherapeutic as well as targeting and visualization functions is anticipated to achieve the integration of tumor diagnosis and treatment in the future. (b) Due to their inherent nature, surface modifiers can be grafted onto the coating surface. However, when melanin is used as an intermediate layer, it is important to consider whether the grafted functional molecules retain their functionality. (c) Currently, melanin-based drug-laden nanoparticles are released at a rate of approximately 20%. Due to the unique structure of MNPs, which can be degraded under micro-acidic tumor conditions to achieve responsive drug release, a thorough analysis of the release behavior of drug-laden melanin nanoparticles is needed to clarify the release process and to explore the appropriate design of melanin-like coatings to enhance the effective and targeted release of adsorbed drugs at tumor sites. (d) No FDA-approved therapeutic, diagnostic nanoparticles have been used as delivery vehicles to date, in part because of the significant heterogeneity in the design and synthesis of these drugs. Biomedical imaging has the potential to encapsulate a variety of therapeutic agents within melanin nanoplatforms. To maximize the clinical translation of MNPs, it is necessary to have a comprehensive understanding of the nanoparticle composition, formulation, shape, surface charge, hydrodynamic diameter, solubility, stability, route of administration, distribution, metabolism, clearance, and potential toxic effects. The majority of nanocontrast agents based on melanin are still in the experimental phase, and although significant progress has been made, few have been evaluated in humans. The continued development of MNPs should concentrate on enhancing targeting specificity while minimizing toxicity. In addition, it cannot be overstated how important it is to improve translational potential by using appropriate animal models, as well as to understand the specific pharmacokinetic profile of these agents in humans.

Regarding in vivo tumor therapeutic applications: (a) As discussed in this review, melanin has potential toxicity in tumor pathological conditions, including the potential to promote immunosuppression of the tumor microenvironment and increase oxidation. The structure–property-function understanding is inadequate due to the lack of research on their structure and polymerization mechanisms. It is necessary to combine experiments and calculations to explore the detailed polymerization mechanisms and formation kinetics involved in the synthesis of MNPs, to understand the undesirable byproducts of the polymerization process, and to find ways to prevent them. (b) Monomer dopamine, a neurotransmitter with similar effects to those of neuropharmaceuticals and anticancer agents, can directly affect the release of the PDA nanoplatform, thereby affecting the therapeutic effect, and it is essential to further investigate the biodegradability and toxicological toxicity of PDANPs. (c) Numerous reports have described the potential of MNPs combined with surface modifiers, but more information on their pharmacokinetics, acute toxicity, and long-term toxicity should be gathered prior to their clinical application. Future research will focus on enhancing the translational potential of these drugs via suitable animal models and elucidating the unique pharmacokinetic profile of these drugs in humans. (d) Although MNPs are promising candidates for clinical PTT, their own photothermal conversion rates are unsatisfactory, and they must be combined with other antimicrobial therapeutic approaches. For one thing, it can be combined with conventional inorganic photothermal materials for synergistic antitumor treatment or utilize photothermal responsiveness to achieve on-demand release of chemicals at the tumor; for another thing, it can be combined with PDT and other integrated tumor treatment platforms, supplemented by PTT to reduce laser density and local photothermal temperature and achieve synergistic antitumor effects. In addition, melanin-based PTT relies on NIR; however, while current technology can precisely control the timing and intensity of irradiation, there is still considerable room for improvement in terms of spatial precision. With the rapid development of fiber optics in the biomedical field, it is anticipated that this newer technology will contribute to the future clinical translation of PTT.

In conclusion, the past decade has seen tremendous progress in the use of MNPs as highly effective diagnostic and therapeutic platforms, and melanin has been proposed as a "molecular target" for targeted anticancer therapies based on the rational design of MNPs that promise to replace conventional contrast agents and photothermal therapeutics [164]. MNPs will accelerate the clinical translation and lead to major commercial breakthroughs through the collaboration of multidisciplinary teams of chemists, pharmacists, biologists, physicists, engineers, and physicians in response to the current challenges facing MNPs.

## Data Availability

Not applicable.

## Abbreviations

MNPs: Melanin nanoparticles
PDA: Polydopamine
PTT: Photothermal therapy
DHI: 5–6-Dihydroxyindole
DAQ: Dopamine quinone
ESC: Embryonic stem cell
HPLC: High-performance liquid chromatography-mass spectrometry
mPEG-NH_2_: Methoxypolyethylene glycol amine
HIF-1: 1-Alpha
SET-LPR: Single-electron transfer activated radical polymerization
SI-ATRP: Surface-initiated atom transfer radical polymerization
RAFT: Reversible addition-rupture chain transfer polymerization
RES: Reticuloendothelial system
PAI: Photoacoustic Imaging
ICG: Indocyanine green
PDT: Photodynamic therapeutic
MRI: Magnetic resonance imaging
RT: Radiotherapy
SRF: Sorafenib
PET: Positron emission computed tomography
CT: Chemotherapy
PDT: Photodynamic therapy
PS: Photosensitizers
FA: Folic acid
